# A two-stage framework for cost-sensitive predictive maintenance using deep learning, GANs, and risk-aware clustering

**DOI:** 10.1038/s41598-026-42910-4

**Published:** 2026-03-21

**Authors:** Ali Hakami

**Affiliations:** Mechanical and Industrial Department, College of Engineering and Computing in Al-Gunfudha, Umm Al-Qura University, 21961 Mecca, Saudi Arabia

**Keywords:** Predictive maintenance (PdM), WGAN-GP, Maintenance scheduling, Clustering, Data scarcity, GAN-based data augmentation, Maintenance optimization, Engineering, Mathematics and computing

## Abstract

Predictive maintenance (PdM) has seen significant advances through machine learning, yet its practical deployment remains challenged by data scarcity, system complexity, and uncertainty in cost-related decisions. The majority of current PdM strategies are concerned with enhancing Remaining Useful Life. Prediction accuracy of (RUL) in isolation and with maintenance scheduling as a problem (secondary or fixed). This study proposes a component based, decision oriented predictive maintenance (PdM) approach that links Remaining Useful Life (RUL) to optimization of maintenance. The two-stage framework proposed anticipates the component-specific RUL prediction where Long Short-Term Memory (LSTM) models was used to predict the Remaining Useful Life (RUL) of individual components. To address sparsity in failure data, Wasserstein Generative Adversarial Networks Gradient Penalty (WGAN-GP) were utilised to fill in run-to-failure sequences, stabilizing downstream modeling. In the second step, similar components in terms of Remaining Useful Life (RUL) degradation are clustered together by Density-Based Clustering Space (DBSCAN), which allows opportunistic maintenance. A decision on maintenance is then optimized cost-conscious grid search which works on fitted RUL distributions and a normalized. Not only based on point RUL estimates, but on a risk proxy. Empirical experimentation across multiple industrial components of a water bottling plant system indicates that the proposed approach continually reduces corrective failures and normalized maintenance costs as opposed to non-clustering approaches such as random choice and fixed choice of maintenance point. Sensitivity analysis also indicates that the optimal maintenance levels be consistent over a vast spectrum of cost assumptions, which emphasizes the resilience of the structure in economic uncertainty. Overall, this study contributes a robust and scalable maintenance literature that combines data augmentation, component based clustering, and risk aware optimization. This helps advance predictive maintenance practices into a more practical, cost aware decisions.

**Supplementary Information:**

The online version contains supplementary material available at 10.1038/s41598-026-42910-4.

## Introduction

Industrial maintenance is an important practice in ensuring reliability and efficiency in production systems, especially in high throughput production systems like water bottling plants^1^. This is because it focuses on reducing unplanned failures that cause severe financial losses, through unanticipated production downtimes, costly repairs, and disruptions in the supply chain^2^. With the advent of Artificial intelligence (AI) such failures can be monitored and avoided This allows industrial systems to remain running in their most productive state thus increasing system throughput and consequently the overall profitability^3^.

Industrial maintenance strategies can be categorized as reactive (run-to-failure), time-based, and condition-based^4^. Reactive maintenance is costly due to unexpected breakdowns^5^, preventive maintenance on the other hand can necessitate unnecessary servicing and added expenses^3^. Predictive maintenance involving the use of advanced technologies such as machine learning (ML) and other data driven techniques, has gained popularity and can help optimize maintenance schedules while minimizing maintenance costs^6–8^.

Predictive maintenance (PdM) incorporates failure prognostics that estimate degradation or Remaining Useful Life (RUL) of industrial components^9^. RUL prediction models use historical failure dataset to determine when a specific machine is most likely to fail, which enable maintenance teams to intervene prior to failure^10^. The advancement in technology and incorporation of artificial intelligence has sparked growing interest in many fields, including PdM^11^.

### Data scarcity as a challenge to PdM

Past research have also shown that new components or systems for instance, in predictive maintenance using machine learning, may have little to no historical data available^12^.The next available set data may be unrepresentative of the normal operation, with limited failure instances^13^. This leads to poor model generalization and poor ability to predict failure^14^.

To overcome this problem, several approaches, including data augmentation, trans- fer learning, and physics-informed models, have been proposed. Data augmentation methods such as bootstrapping and SMOTE (Synthetic Minority Over-sampling Tech- nique) have been employed to artificially increase failure samples^15,16^ while transfer learning has helped models learn from related domains where there maybe abundant failure data^17^.

One augmentation strategy that has received much attention in recent years is Generative Adversarial Networks (GANs). GANs model features a generator model, and a discriminator model, the two work in a competitive way, where the generator generates fake failure trajectories, and the discriminator learns to differentiate between the real data and fake data. In this context, GANs mimic failure regions and produce synthetic data with inherent characteristics of real failure patterns^12^. Recent studies have demonstrated that data augmentation utilizing Generative Adversarial Network can enhance the resilience of ML models in industrial settings.

For instance^18^ proposed a novel approach, called Scarcity-GAN, to data limitations. Their model used external datasets of defect features and an encoder-decoder network. The comparison of the proposed augmentation methods with other existing augmentation methods for industrial datasets pointed to higher accuracy.

^19^presented an approach which employed Conditional Wasserstein GANs (CWGANs) to generate syn- thetic spectral data. The study also revealed that conditioning WGANs for proper labeling and avoiding mode collapse helped to enhance the performance of the model. The CWGAN also showed that the use of images and augmentation of Mel-Frequency Cepstral Coefficients (MFCCs) enhanced a feed-forward neural network for classification, thereby highlighting the general applicability of GANs for more than image generation tasks.

Vilela-perez^20^ presented the EffBaGAN architecture, a GAN-based augmentation model designed to mitigate the issues of data scarcity and class imbalance in RS. This paper proposes an EfficientNet-based discriminator and generator developed on BAGAN to help improve the computational efficiency of the model. When tested on multispectral vegetation images, EffBaGAN has increased in efficiency both in training and testing compared to vision transformers and Residual BAGAN, making it significantly accurate for data sets with limited data samples. Hakami^12^ addressed data scarcity in predictive maintenance (PdM) by leveraging GANs to generate synthetic run-to-failure data, improving ML model training. Their findings highlighted the importance of GANs in data augmentation in PdM. Many other researchers, including^21–24^ and^25^, have successfully applied GANs for data augmentation in various fields, such as disease diagnosis, pharmacogenetics, Synthetic Tabular Data Generation (STDG) and solid rocket motors (SRMs) remote sensing. However, the challenge is not only the limited use of data augmentation techniques, but the absence of frameworks that explicitly link augmented data to robust maintenance decision-making. Existing PdM studies that employ GANs or other augmentation methods primarily focus on improving Remaining Useful Life (RUL) prediction accuracy, often treating prediction as an isolated objective. As a result, little attention is given to how augmented data influences downstream tasks such as component grouping, maintenance coordination, and cost-aware scheduling under uncertainty.

In this work, component-specific GANs are used not merely to increase data volume, but to stabilize RUL distributions in the presence of severe failure sparsity. This enables reliable clustering of components with similar degradation states and supports risk-aware optimization of maintenance thresholds. By integrating data augmentation with decision-oriented clustering and scheduling, the proposed framework moves beyond prediction-centric PdM toward a coordinated, component-level maintenance strategy.

### Clustering maintenance practices

The primary goal of maintenance strategies is to minimize unnecessary interventions which add on to the costs while at the same time avoid failure-related costs that arise from downtime. Over-maintenance leads to performing unnecessary repair and replacement of parts, expenses in labor, and inactivity time, while under- maintenance leads to system failures, reduction in system throughput, and high downtime expenses. In response, several clustering techniques have been employed in various maintenance settings for decision-making enhancement. The purpose of clustering is to categorize the components or the maintenance tasks for a particular system based on the degradation pattern, operational condition or failure risk and hence flexibility in the scheduling and resource allocation. In certain industries, clustering has been applied to similar but related idea covered under condition-based maintenance, where assets are grouped according to wear patterns to improve modeling. For instance, proposed the genetic algorithm for clustering in maintenance scheduling related to healthcare facilities with improved asset performance and high operational availability.

Additionally, pointed that clustering maintenance actions in manufacturing systems which failure modes take into consideration three and move toward the perfect maintenance action centralized type influence was seen to lower cost in relation to the frequent rate of failure as well as a time optimization. They also stress on the utility of using clustering to reduce on the maintenance costs and production loss as high- lighted by where the authors write on the identification of phases in algorithms of TVIPs specifically when using clustering to monitor production assets whenever fault annotated databases for supervised learning models are scarce. Their work utilised a new method called Latent Dirichlet Allocation for clustering of applications in maintenance. Further elaborate on early-stage maintenance clustering and its effectiveness on maintenance planning. The literature analysis together with the case studies used in the study showed that gains made through clustering in identification phase were

4.6 per cent of the planned work hours while the gains made in the planning phase were 2.7 per cent and 2.4 per cent in the scheduling phase. Especially, the findings highlight the positive aspects of early clustering in terms of eradicating wasteful work, while calling for further study on the issue. Yet, clustering has not been a subject of extensive research in PdM. Most of the works use a global approach, where the goal is to train models to predict failure probability or Remaining Useful Life (RUL) for the overall system. This perspective fails to consider that there are different RULs associated with different components. In addition, some parts might be maintained more often than required, while others may not be maintained when they should be, and this leads to ineffectiveness. For example focused on the significance of each individual RUL estimations in multi-component systems to minimize the costs of equipment upkeep. Likewise, analysing the case of turbofan engines, showed that incorporating probabilistic RUL prognostics in maintenance planning enhances the capacity to curb costs compared to non-probabilistic time-based policies. Also, studies have focused on the issues with the system-level prognosis and stressed the importance of considering the state of the each component as well as their interaction with one another to improve the accuracy and efficacy of predictions. From these con- siderations, this work introduces a localized maintenance scheduling strategy where components are grouped according to their RULs. Hence, putting similar components with similar failure profiles in a group will ensure that maintenance effort is focused on specific components, leading to reduced overall cost through minimizing time lost and efficient utilization of resources.

Beyond improving prediction accuracy, an important objective of predictive maintenance is to enable coordinated and cost-efficient maintenance actions across system components. In practice, maintaining components independently based solely on individual RUL estimates can lead to redundant interventions, unnecessary downtime, or missed opportunities for joint servicing. By explicitly grouping components with similar degradation states using RUL-based clustering, the proposed framework enables opportunistic maintenance, whereby components that are likely to require service within a similar horizon can be maintained together. This approach contrasts with existing PdM strategies that either assume homogeneous component behavior or apply global maintenance thresholds without coordination. As a result, the proposed clustering-based strategy supports more flexible, interpretable, and economically efficient maintenance planning at the component level.

## Experimental methods

### Data collection and augmentation

This research aims at analysing run-to-failure data obtained at one-hour time intervals from a water bottling plant with 16 components. The system was observed for a total of 3,319 operating hours; data collected includes throughput, defined as gross and net number of bottles produced, failure time and type if any, line speed, bottle size, and machine/component code. These recordings were carried out by the production engineers at the different machine stations on the production line. In an effort to ensure that the system components are not overtly named or de-identified, the component names were coded as M1,M2,...,M16.

Prior to model training, the raw maintenance logs were processed to obtain consistent machine-level time series at an hourly resolution. Planned maintenance activities (e.g., CIP changeovers) were excluded, as these operations are deterministic and do not reflect stochastic degradation behavior. Since failures were recorded for different machines independently, the data were processed separately for each machine, with system-level events involving multiple machines included in the data of each affected component.

For each machine, hourly records were segmented into run-to-failure sequences, where a run is defined as the period between two consecutive failure events. Run lengths are therefore variable and non-deterministic, reflecting the stochastic nature of component degradation, as summarized in Table 1. For simplicity, it was assumed that every time a component (machine) failed, it was fully restored, hence failures on the previous runs does not affect operations on the next run. This defines the independence between the runs.

**Table 1:** Run statistics for machine components.

Machine component	Total runs	Min run (hrs)	Max run (hrs)	Mean run (hrs)	Median run (hrs)
M0	594	1	427	5.49	1
M1	612	1	130	5.33	3
M2	223	1	222	14.62	2
M3	161	1	202	20.25	5
M4	104	1	340	31.35	2
M5	29	1	642	112.41	7
M6	19	1	541	171.58	99
M7	27	1	936	120.74	7
M8	18	2	657	181.11	116
M9	3	549	1589	1086.67	1122
M10	2	1106	2154	1630.00	1630
M11	2	1237	2023	1630.00	1630
M12	2	843	2417	1630.00	1630
M13	2	714	2546	1630.00	1630
M15	2	552	2708	1630.00	1630
M15	2	193	3067	1630.00	1630

A set of degradation-relevant features was selected, including failure duration, line speed, product SKU (bottle volume), age at repair (approximated by the midpoint for ranged values), and a derived throughput deficit feature computed as the difference between gross and net production. All features were normalized using min–max scaling on a per-machine basis to ensure comparability and to prevent information leakage across components.

**Fig. 1:**
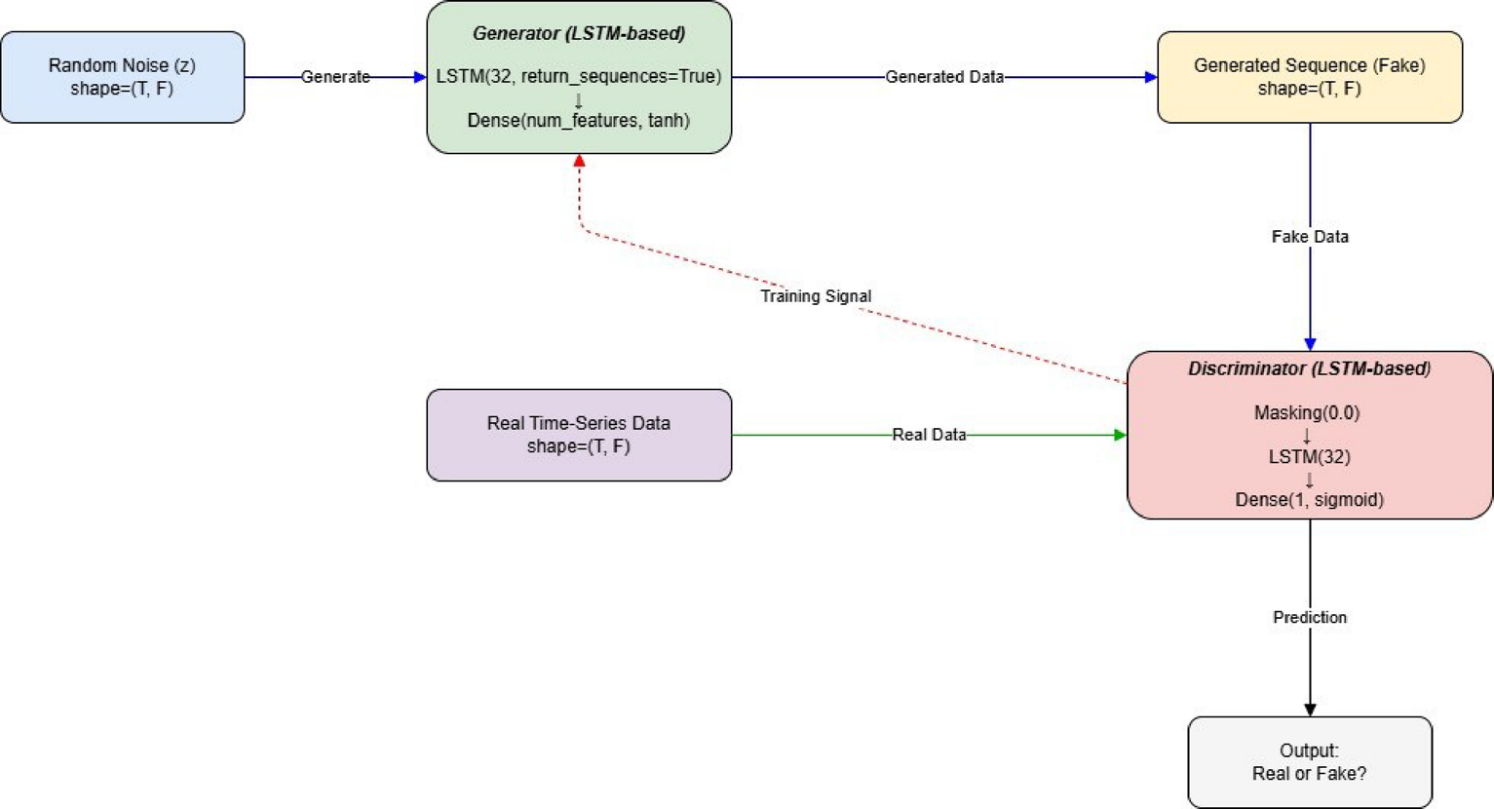
The structure of GANs model.

A common problem in PdM datasets is data sparsity, especially where failures are rare in some components. For example, components M9 to M15 had at most 3 runs, ability of learning-based models to capture degradation patterns. To get around this issue, data augmentation was performed using a Wasserstein Generative Adversarial Network with Gradient Penalty (WGAN-GP) trained separately for each machine with sufficient failure history. The WGAN-GP learns the distribution of machine-specific run-to-failure sequences and generates additional synthetic runs with similar temporal and statistical characteristic. The WGAN-GP learns the distribution of machine-specific run-to-failure sequences and generates additional synthetic runs with similar temporal and statistical characteristics. The Lipschitz constraint was enforced using a gradient penalty term with coefficient λ = 10 and the critic was updated five times for each generator update to ensure stable training. Due to the variable and potentially long sequence lengths, a batch size of one was used during training to manage computational cost. Models were trained for up to 100 epochs with early stopping (patience = 5) based on convergence of the Wasserstein objective. To control the extent of augmentation, a fixed ratio *k* = 15 was used, meaning that for each observed run-to-failure sequence, 15 synthetic sequences were generated. The synthetic and real sequences were then combined to form augmented training sets, improving robustness while preserving machine-specific degradation behavior. Figure 1 visualizes the GAN workflow.

### Remaining useful life (RUL) prediction model

To estimate the RUL of each component, the Long Short-Term Memory (LSTM), a recurrent neural network specialized for sequential, was utilized. In this context it was intended to capture temporal dependencies and failure deceleration patterns by using historical failure sequences. The models operate on variable-length run-to-failure sequences. Each model(one per machine) operate on variable-length run-to-failure sequences and accepts multivariate time-series inputs and applies a masking layer to handle padded time steps arising from variable-length sequences. The masked inputs are processed through one or more stacked LSTM layers, all configured to return full sequences, followed by a linear dense output layer that predicts RUL at each time step.

Due to heterogeneity in degradation behavior and data availability across components, RUL models were trained independently for each machine using a common architectural template with per-machine hyperparameter tuning. The tuning process explored architectures with one to three LSTM layers and 64 to 256 units per layer, as well as learning rates between ${10}^{-3}$ and ${10}^{-4}$. For each machine, a limited random search of 5 trials each of 20 epochs was conducted to select the best-performing configuration based on validation loss. Model training was performed for up to 100 epochs using the Adam optimizer, with early stopping applied (patience = 10) to mitigate overfitting. A custom loss function was used, combining custom mean squared error with monotonicity and trend penalty terms to enforce physically consistent, non-increasing RUL trajectories over time while respecting padded regions of the sequences. Validation loss was monitored throughout training to guide model selection and convergence.

### Probability estimation of failure

To quantify degradation and failure risk for each component, parametric probability distributions were fitted to the predicted Remaining Useful Life (RUL) values obtained from the LSTM models. The candidate distributions were restricted to forms commonly used to model lifetime and degradation behavior (Weibull, exponential, gamma, lognormal, inverse Gaussian, Gompertz, and fatigue-life), and the best-fitting distribution for each component was selected based on goodness-of-fit criteria.

Most importantly fitting distributions to predicted RUL values does not directly yield failure-time probabilities. Instead, the fitted distribution characterizes the empirical distribution of RUL magnitudes, where larger values indicate healthier operation and values approaching zero indicate proximity to failure. To account for this, a normalized CDF-based survival index was defined as a proxy for relative health and failure risk.

Let ${f}_{i}(t)$ denote the probability density function (PDF) of the fitted RUL distribution for component iii, and.

Let

1 $${F}_{Ri }({t}_{k}) ={\int }_{0}^{{t}_{k }}{f}_{i}(t ) dt$$

be the corresponding cumulative distribution function (CDF). Since failure corresponds to RUL = 0, the probability mass at or below the failure boundary is given by ${F}_{Ri }(0)$. The proposed survival index at horizon ${t}_{k}$ is defined as:

2 $${SI}_{i }\left({t}_{k}\right)=\frac{{F}_{Ri }\left({t}_{k}\right)-{F}_{Ri }(0) }{{1-F}_{Ri }(0)}$$

which represents the normalized probability mass between the failure boundary and the predicted RUL value. This index increases monotonically as the predicted RUL moves farther from zero and is bounded in the interval [0,1].

A corresponding relative failure risk proxy is then defined as

3 $${Risk}_{i }\left({t}_{k}\right)=1-{SI}_{i }\left({t}_{k}\right)$$

This representation gives a numerically stable distribution-sensitive measure of distance to failure, without a real failure-time representation. The resulting risk proxy is then adopted to aid clustering and cost-conscious maintenance decision-making to facilitate planning, which takes into account relative degradation severity, but not the point estimates of RUL individually.

### Maintenance scheduling strategy

In order to reduce the cost of maintenance and enhance efficiency, we will implement a two-stage, component-based maintenance schedule approach based on distributions of the RUL (Remaining Useful Life) that is predicted.

#### Stage 1: Clustering based on RUL proximity

We initially cluster components with statistically similar degradation pattern (RUL predictions) instead of using a global predictive maintenance (PdM) rule to all components. It is used to cluster together components whose remaining lifetimes are similar, and not have to waste time servicing components that are in dissimilar health conditions.

Let *C*_1_ ,*C*_2_...*C*_*k*_ be the resulting clusters from a similarity-based grouping of components based on predicted RULs *RÛL*_*i*_ where each component *i* ∈ *C*_*k*_ . We define a proximity threshold parameter *δ*, which determines the distance between RULs for components to belong to the same cluster:

4 $$\left| {R\hat{U}L_{i} - R\hat{U}L_{j} } \right| \le \delta ,\;\forall i,\;j \in C_{k}$$

This threshold *δ* is an important optimization variable, it determines the number of granules in a clustering and consequently, it influences the scheduling and costs obtained. Our implementation availed Density-Based Spatial Clustering of Applications with Noise (DBSCAN), in which the number of clusters is not specified, which automatically identifies noise and outlier elements that are unsuitable to maintenance coordination, and cluster formation on the basis of RUL space proximity. According to this formulation, the threshold d is equal to the e parameter and it determines the maximum distance that can exist between the elements of the same cluster.

#### Stage 2: Optimal maintenance timing via grid search

After creating clusters, we calculate the maintenance RUL threshold *t*_*k*_ per cluster *C*_*k*_ to be minimum of the expected cost of servicing.

### Cost components

The total expenditure incurred in maintaining a cluster *C*_*k*_ at RUL threshold *t*_*k*_ has the following:

- **Preventive maintenance cost** *C*_*p*_: Cost incurred when a component is serviced before reaching the failure boundary.
- **Corrective (failure) maintenance cost** *C*_*f*_: Higher cost associated with unplanned repairs and interventions related to failure.
- **Downtime cost** *C*_*d*_: Cost related to lost production output due to component failure or maintenance downtime.

Let *r*_*i*_(*t*_*k*_) be the RUL-based failure risk proxy for component $i \in C_{k}$ evaluated at the maintenance RUL *t*_*k*_, the expected maintenance cost for the cluster is computed using this risk proxy to weight corrective and downtime costs.

5 $$\mathrm{Cos} t\left( {C_{k} ,t_{k} } \right)\sum\limits_{{i \in C_{k}}} {\left[ {r_{i} \left( {t_{k} } \right) \cdot \left( {c_{f} + c_{d} } \right) + \left( {1 - r_{i} \left( {t_{k} } \right)} \right) \cdot C_{p} } \right]}$$

$$i \in C_{k}$$

The objective is:

subject to:

6 $$Min_{{\delta ,\;t_{1} ,\;t_{2} ...\;t_{k} }} \sum\limits_{{k - 1}}^{K} {\mathrm{Cos} t} (C_{k} ,t_{k} )$$

- $$\left| {R\hat{U}L_{i} - R\hat{U}L_{j} } \right| \le \delta ,\;\forall i,\;j \in C_{k}$$
- *r*_*i*_ (*t*_*k*_) ≥ *θ* , where θ is a predefined risk threshold triggering maintenance.
- *t*_*k*_ ≤ *min* $i \in C_{k}\, {R\hat{U}L_{i} }$ *RU*^ˆ^*L*_*i*_ to prevent maintenance beyond the earliest predicted failure within the cluster.

Throughout our maintenance scheduling approach, we adopted a grid search to establish the best threshold *t*_*k*_ of every cluster of components with similar Remaining Useful Life (RUL) distributions. This was due to the nature of the anticipated cost function which is usually a piecewise constant then monotonic function. RUL-based failure risk proxy on small values of tk probability of failure r_i_(*t*_*k*_) is almost zero, which leads to a flat cost region, in which the overall cost is about the same as the cost of preventive maintenance. As *t*_*k*_ gets closer to the critical region of the RUL distribution, r_i_(*t*_*k*_) starts to increase at a high rate which results in the cost suddenly soaring because of the danger of failure and the resultant downtime. Due to this phenomenon, the classical optimization tools such as the gradient descent cannot be used- they can become trapped in the flat areas or cannot recognize the steep cost rise.

Instead, grid search methodically considers a finite set of candidate *t*_*k*_ values in the range of observed UR, with which we can determine the best maintenance threshold without utilizing gradients. When there is a set of candidate *t*_*k*_ values which produce the same minimal cost we select the largest one and delay maintenance as long as we can without exposing ourselves to higher risk. We also have restrictions to prevent premature and late maintenance, i.e. *t*_*k*_ should not be greater than the earliest noted RUL within the cluster and the probability of failure should be less than a given cut-off.

#### Stage 3: Scheduling maintenance time per cluster

**Fig. 2:**
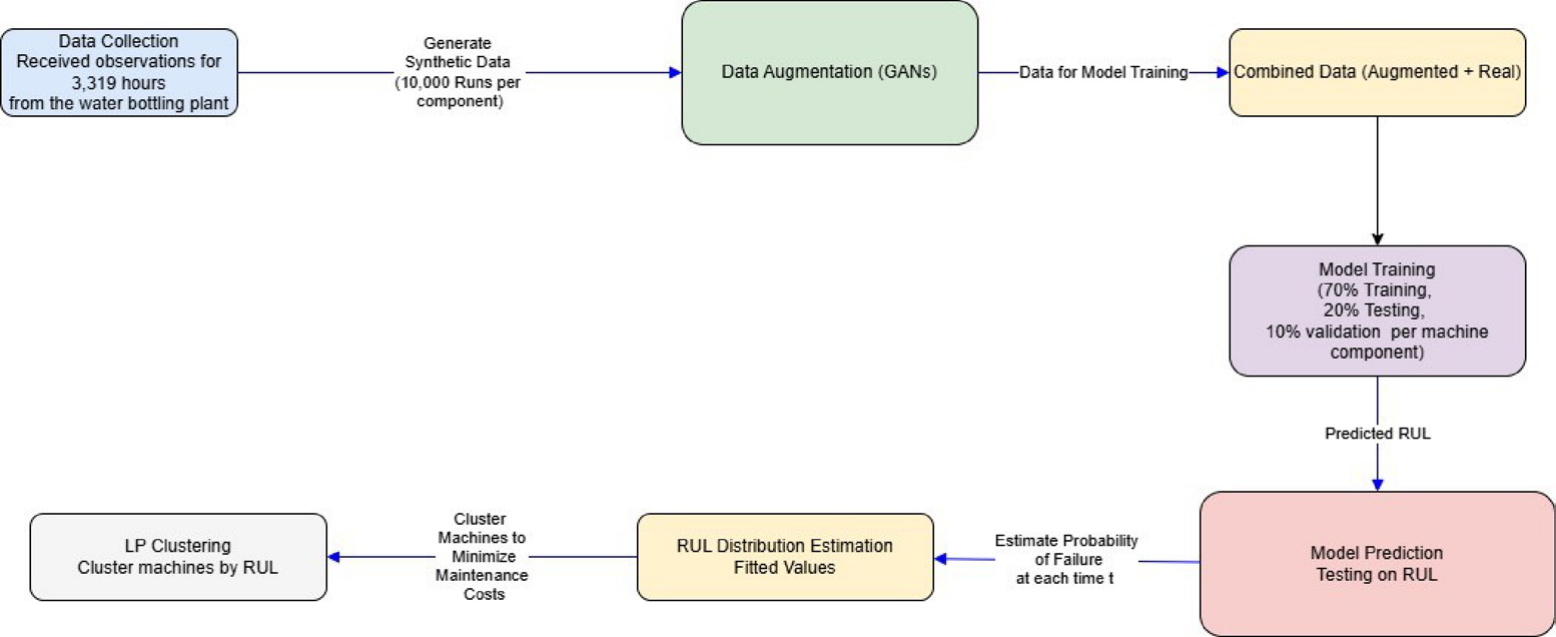
The Conceptual framework.

Then, for every cluster *C*_*k*_, the optimal maintenance RUL threshold *t*_*k*_ is chosen such that it decreases the overall expected cost of maintaining all the components of the cluster to the least. This threshold is not fixed a priori but an optimized based on different factors. This represents the RUL between the potential for performing early, possibly unnecessary preventive maintenance and when the gear could fail with subsequent downtime. The chosen threshold *t*_*k*_ makes it possible to estimate the total risk of failure of components in the cluster until a given time point, thereby successfully moderating the identified risks. This means that after calculating candidate maintenance thresholds for all units in a feasible range (from the current time to the maximum RUL within the cluster), the model identifies the point that leads to the minimum total cost of preventive and failure cost for the entire cluster. It enables systematic planning and efficient execution of maintenance so as to reflect the state of degradation of parts involved in the system. Figure 2 below illustrated the conceptual framework of this proposed methodology.

## Results

### Synthetic data generation using GANs

Since failure sequences are grossly scarce for each of the components, WGAN-GP was trained separately on each of the machine component dataset for up to 100 epochs with a batch size of 1. To overcome cases of overfitting and improve training rate, early stopping was applied based on Wasserstein critic distance, with 10 epochs of patience to allow model training to stop as soon as the performance stagnates. Training history curves for Machines 1, 2, and 3 are shown in Appendix A, Figs. A1–A3. Illustrating the typical convergence behavior observed across most of the models, where the generator and critic loss shows a rapid initial increase/decrease followed by slower progression as training advance. This slowdown indicates that the critic became learned to distinguish between real and synthetic data, while the generator simultaneously learned to generalize and come up with new sequences having statistical properties similar to the real data. The complete set of training plots for all 16 machines is provided in Supplementary file S1.

**Fig. 3:**
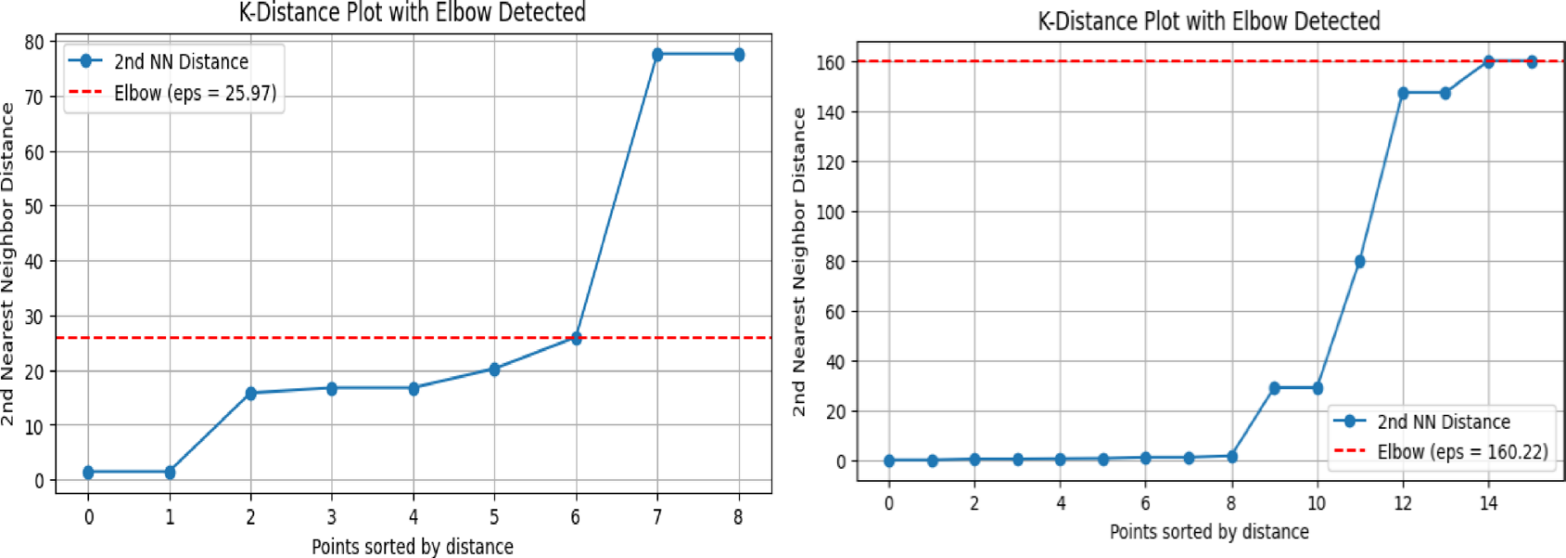
Optimal threshold for cluster dissimilarity (*δ*).

Moreover, each feature on the generated data were compared with their corre- sponding feature on the real data using kernel density estimate graphs(KDE).The comparisons show exactly superimposable distributions of the features in the real and synthetic data, Appendix B, Figs. B1–B3 show these comparisons for Machine 0-3. The complete set for all the 16 machines is presented supplementary file S1. PCA plots were also used to visualize similarity between the synthetic and the real data in a lower dimension. Appendix C shows these comparisons for the first 3 machines.

Kolmogorov–Smirnov (K–S) tests were conducted to evaluate the goodness of fit between real and generated feature distributions for each machine component. While the K–S tests were statistically significant for all components, this does not imply poor synthetic data quality. Such tests are highly sensitive-especially in the tails and large samples—and may not reflect practical or perceived similarity. Therefore, they should be complemented with visual and model-based evaluations, which in this case support the utility of the generated data. Table 2 below reports the K–S test results for real vs. synthetic data across different machines and features.

**Table 2:** K–S test results for real vs. synthetic data by machine and feature.

Machine	Feature	K–S Statistic	*p*-value
M0	Feature 0	0.9473	< 0.001
	Feature 1	0.7622	< 0.001
	Feature 2	0.4707	< 0.001
M1	Feature 0	0.9913	< 0.001
	Feature 1	0.8177	< 0.001
	Feature 2	0.6043	< 0.001
M2	Feature 0	0.9959	< 0.001
	Feature 1	0.7128	< 0.001
	Feature 2	0.5150	< 0.001
M3	Feature 0	0.9628	< 0.001
	Feature 1	0.8274	< 0.001
	Feature 2	0.5670	< 0.001
M4	Feature 0	0.9974	< 0.001
	Feature 1	0.8405	< 0.001
	Feature 2	0.4980	< 0.001
M5	Feature 0	0.9941	< 0.001
	Feature 1	0.7702	< 0.001
	Feature 2	0.4834	< 0.001
M6	Feature 0	0.9752	< 0.001
	Feature 1	0.8979	< 0.001
	Feature 2	0.4933	< 0.001
M7	Feature 0	0.9965	< 0.001
	Feature 1	0.8740	< 0.001
	Feature 2	0.5295	< 0.001
M8	Feature 0	0.9275	< 0.001
	Feature 1	0.8084	< 0.001
	Feature 2	0.4732	< 0.001
M9	Feature 0	0.9894	< 0.001
	Feature 1	0.7009	< 0.001
	Feature 2	0.4909	< 0.001
M10	Feature 0	0.9934	< 0.001
	Feature 1	0.7229	< 0.001
	Feature 2	0.4667	< 0.001
M11	Feature 0	0.9276	< 0.001
	Feature 1	0.8005	< 0.001
	Feature 2	0.5679	< 0.001
M12	Feature 0	0.9928	< 0.001
	Feature 1	0.7135	< 0.001
	Feature 2	0.4826	< 0.001
M13	Feature 0	0.9774	< 0.001
	Feature 1	0.8131	< 0.001
	Feature 2	0.7242	< 0.001
M14	Feature 0	0.9454	< 0.001
	Feature 1	0.7269	< 0.001
	Feature 2	0.5262	< 0.001
M15	Feature 0	0.9867	< 0.001
	Feature 1	0.7558	< 0.001
	Feature 2	0.8348	< 0.001

### LSTM-based remaining useful life (RUL) prediction performance

For predicting Remaining Useful Life (RUL) of each component, we developed 15 different Long Short-Term Memory (LSTM) models. Hyperparameters were fine-tuned including the number of LSTM units between 32 and 256, use of single LSTM layer and stacked LSTM layers, and the optimization algorithm between Adam and RMSprop. To prevent overfitting, dropout regularization rates between 0.0 and 0.4 were also included. The models were trained with epochs ranging of up to 100. However, an early stopping callback was added to the model training to halt model training if the model validation loss does not improve for 10 consecutive epochs. Training history curves for Machines 1, 2, and 3 are shown in Appendix D, Figs. D1–D3. The complete list of model training history is available on Supplementary S1. Generally, the graphs show stability of training without overfitting.

To evaluate the efficiency of the RUL prediction models, five metrics were calculated in each test set. Mean Absolute Error (MAE), Root Mean Square Error (RMSE), Median Absolute Error (Median AE), Mean Absolute Percentage Error (MAPE), and the percentage of predictions falling within ±10 cycles of the true RUL (Within ±10). To assess the impact of data sparsity and the contribution of GAN-based augmentation, model performance is reported both for models trained with synthetic data augmentation and for models trained using original data only. The results are as shown in Table 3 below:

**Table 3:** Evaluation metrics for RUL prediction models.

Machine	Loss	MAE	MAPE	MSE	${R}^{2}$	RMSE	Within ± 10 (%)	Median AE
*With GANs*								
M0	0.0000	0.0012	42,034.3	0.0000	0.9998	0.0044	96.9939	0.0009
M1	0.0017	0.0092	502,903.5	0.0018	0.9829	0.0386	91.6515	0.0058
M2	0.0012	0.0139	286,641.2	0.0012	0.9884	0.0331	86.6526	0.0111
M3	0.0007	0.0083	146,643.6	0.0007	0.9933	0.0237	92.0503	0.0057
M4	0.0009	0.0124	190,514.8	0.0008	0.9919	0.0270	87.5009	0.0099
M5	0.0106	0.0251	83,693.4	0.0031	0.9681	0.0536	80.8075	0.0154
M6	0.3740	0.2490	359,502.9	0.0960	−0.0241	0.3099	7.4971	0.2094
M7	0.5357	0.2310	423,743.1	0.0700	0.2639	0.2645	7.9589	0.2338
M8	0.3927	0.2708	161,375.6	0.1039	−0.0289	0.3224	3.7097	0.2510
M9	0.3243	0.3074	162,346.3	0.1405	−0.6839	0.3748	4.9276	0.2478
M10	0.2637	0.2810	150,411.9	0.1144	−0.3717	0.3383	6.5769	0.2500
M11	0.2573	0.2815	159,076.8	0.1148	−0.3768	0.3389	6.5332	0.2502
M12	0.2594	0.2680	151,588.7	0.1014	−0.2153	0.3184	7.3714	0.2501
M13	0.2849	0.2777	131,496.7	0.1111	−0.3323	0.3333	6.7622	0.2500
M14	0.2755	0.2670	136,524.5	0.1004	−0.2036	0.3168	7.4594	0.2501
M15	0.3181	0.2770	107,602.5	0.1104	−0.3245	0.3323	6.8090	0.2500
*Without GAN*								
M0	0.0157	0.0401	7,300,200.00	0.0090	−0.4557	0.0767	5.6829	0.0253
M1	0.2859	0.1983	37,222,690.00	0.1957	39.2678	0.3172	3.4828	0.0827
M2	0.0839	0.1788	2,203,169.00	0.0718	0.8372	0.2680	3.6830	0.1103
M3	0.0561	0.1364	2,175,237.00	0.0369	0.7321	0.1920	2.6899	0.0883
M4	0.0807	0.1550	3,417,938.00	0.0389	0.8222	0.1972	4.5455	0.1187
M5	0.4147	0.5007	521,470.50	0.3914	0.7388	0.6256	4.7288	0.5345
M6	0.1896	0.3276	313,365.90	0.1591	−0.4335	0.3989	2.2082	0.3179
M7	0.0846	0.1871	368,401.30	0.0631	0.5320	0.2512	5.4106	0.1401
M8	0.0709	0.1684	1,604,277.00	0.0476	−0.3390	0.2181	2.3810	0.1063
M9	0.5802	0.6182	117,424.00	0.5387	−2.2174	0.7340	1.6992	0.6268
M10	0.0313	0.1004	157,557.10	0.0143	0.3477	0.1198	13.6528	0.0999
M11	0.0531	0.1112	132,848.60	0.0174	0.4435	0.1317	15.3597	0.1082
M12	0.5912	0.6270	345,774.00	0.5294	0.2290	0.7276	14.1084	0.6499
M13	0.9756	0.8272	424,091.20	0.8753	0.1762	0.9356	15.1610	0.8665
M14	1.4531	0.9551	400,574.90	1.2435	0.3822	1.1151	14.4018	0.9934
M15	7.5799	2.0631	331,461.00	6.5399	0.6924	2.5573	22.3997	1.9799

These results demonstrate substantial variability in predictive performance across machines and between augmented and non-augmented training regimes. Models trained with GAN-based data augmentation generally achieve lower absolute errors, higher Within ± 10 percentages, and more stable values across most components, particularly for machines with limited failure histories. In contrast, models trained without augmentation exhibit pronounced degradation in predictive performance for several machines, including inflated error metrics and negative ${R}^{2}$ values, indicating poor generalization under severe data sparsity. Elevated Mean Absolute Percentage Error (MAPE) values are observed for some components, especially those operating at large RUL scales, where small absolute deviations result in disproportionately large percentage errors. Importantly, within the proposed framework, RUL predictions are not used directly as point estimates for maintenance decisions. Instead, RUL values are normalized and transformed into a distribution-aware risk representation prior to clustering and scheduling. Consequently, downstream decisions rely on relative proximity and risk ranking rather than raw RUL magnitudes. The implications of prediction uncertainty on clustering robustness and cost-aware maintenance optimization are further discussed.

### RUL probability estimation

Following RUL prediction using the LSTM models, parametric distributions were fitted to the predicted Remaining Useful Life (RUL) values of each component in order to characterize the empirical distribution of RUL outcomes across runs. Candidate distributions were restricted to forms commonly used to model lifetime and degradation behavior, including Weibull, exponential, gamma, lognormal, inverse Gaussian, Gompertz, and fatigue-life distributions. For each component, the best-fitting distribution was selected based on the sum of squared error (SSE). Table 4 reports the selected distributions and their estimated parameters for models trained with and without GAN-based data augmentation. When trained with augmentation, the fitted distributions exhibit stable parameter estimates and consistent distributional forms across components. In contrast, models trained without augmentation often result in highly skewed or extreme parameter values, reflecting the effects of severe data sparsity and limited failure observations. It is important to emphasize that these fitted distributions describe the distribution of predicted RUL values, rather than failure-time processes. As such, the estimated parameters are not interpreted directly in a reliability-theoretical sense. Instead, the fitted distributions are subsequently used to derive a normalized, CDF-based RUL risk proxy that quantifies proximity to the failure boundary. This formulation enables robust downstream clustering and maintenance optimization without relying on explicit failure-time or hazard-rate assumptions. The complete list of candidate distributions and fitted parameters is provided in Supplementary File S1.

**Table 4:** The best distributions for each component. See supplementary file S1 for the list of all candidate distributions.

Component	Best_distribution	Param_c	Param_loc	Param_scale	Param_mu	Param_s
*With Gans*						
0	weibull_min	2.05	−29.19	267.71		
1	weibull_min	1.91	−5.86	77.08		
2	weibull_min	1.77	−8.94	127.52		
3	invgauss		−50.92	504.42	0.2923	
4	weibull_min	1.82	−5.55	192.09		
5	weibull_min	2.63	−143.02	505.24		
6	weibull_min	28,829.57	−832,280.17	832,457.97		
7	lognorm		−7502.50	7876.75		0.0093
8	lognorm		−20,092.36	20,261.05		0.0037
*without Gans*						
0	gompertz	0.03	3.58	2.81		
1	lognorm		3.89	1.38	0.420924	
2	weibull_min	1.02	5.74	1.82		
3	lognorm		−2.46	7.11	0.312943	
4	lognorm		−26.40	38.98	0.3843	
5	expon		4.60	0.14		
6	weibull_min	0.30	10.25	32.66		
7	weibull_min	1.42	−2.90	31.10		
8	invgauss		−28.71	137.25		0.3414
9	weibull_min	0.97	33.41	99.20		
10	weibull_min	93,951,780.00	−6,371,177,000.00	6,371,178,000.00		
11	weibull_min	367,215.50	−36,535,740.00	36,536,480.00		
12	lognorm		−32,198,030.00	32,199,230.00	0.000005	
13	lognorm		−33,499,010.00	33,500,700.00	0.000009	
14	lognorm		−33,546,850.00	33,548,210.00	0.000012	
15	weibull_min	210,232,100.00	−106,069,800,000.00	106,069,800,000.00		

### Maintenance optimization strategy

Failure Probability Estimation To address the issues of excessive, planned and forced, as well as excessive and unplanned, maintenance costs we devised and built an efficient and optimised maintenance planning model as described in our experimental methods section:

#### Stage1: RUL-based component clustering

**Fig. 4:**
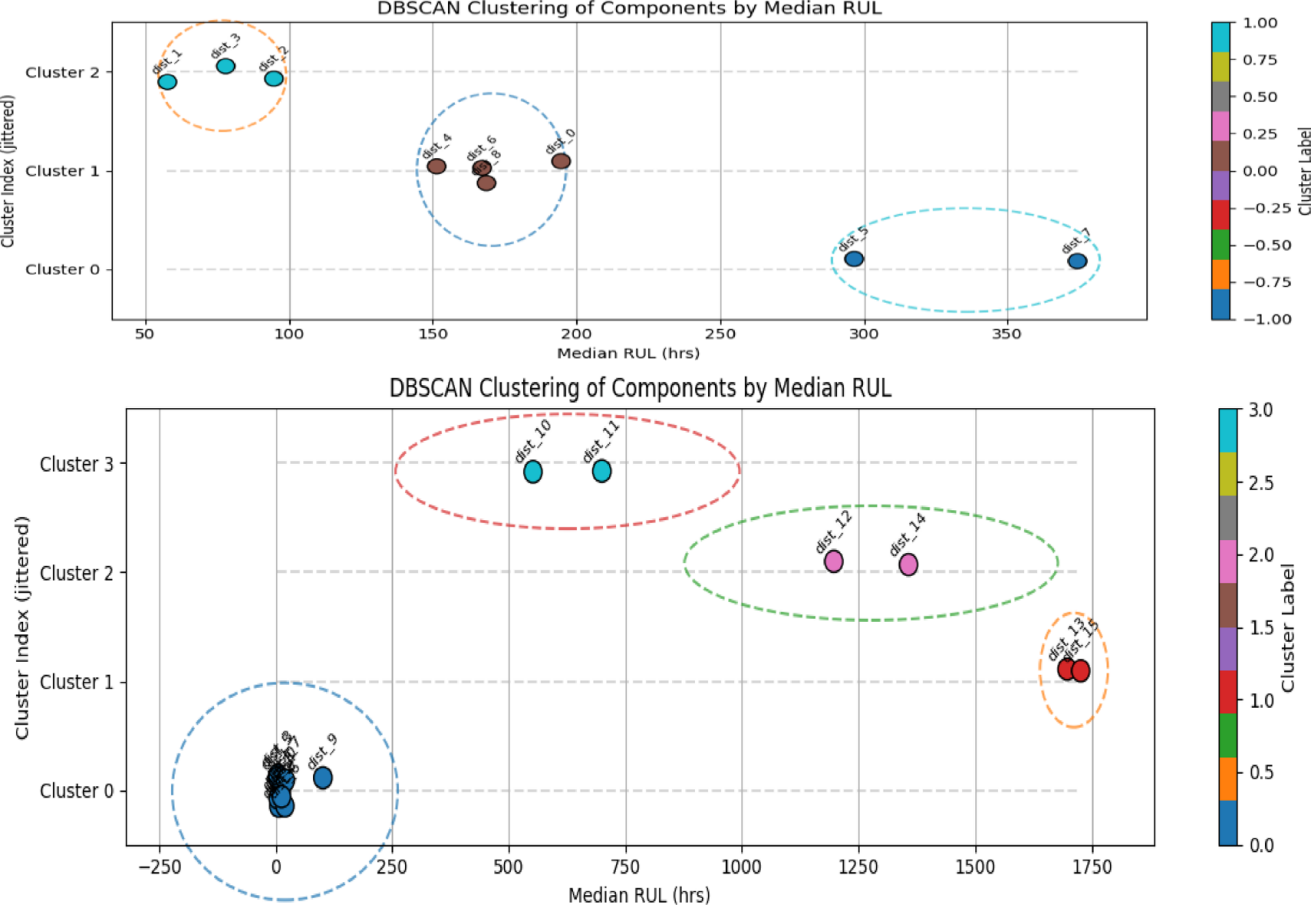
Visual representation of the clusters.

To mitigate excessive preventive maintenance as well as costly unplanned failures, an optimized maintenance planning framework was developed as described in the Experimental Methods section. The first stage of this framework groups components with similar degradation states using predicted Remaining Useful Life (RUL) information, enabling opportunistic maintenance of components with comparable health conditions. Although the dataset comprises 15 components, clustering was performed on the subset of components for which stable RUL distributions could be reliably estimated; the remaining components, affected by extreme data sparsity or unstable distribution fits, were excluded from clustering and scheduled independently. Clustering was carried out using Density-Based Spatial Clustering of Applications with Noise (DBSCAN), applied to the median predicted RUL values. Components classified as noise—i.e., those not sufficiently close to others in RUL space—were treated as single-component clusters and scheduled independently. The maximum allowable distance between median RUL values for components within the same cluster (ε) was selected using the k-distance elbow method. Figure 3 illustrates the k-distance plots and the identified elbow points used to determine ε for both augmented (left) and non-augmented data (right). Based on this analysis, an ε value of 65.14 hours was selected for clustering. Figure 4 visualizes the resulting cluster memberships and relative proximity between components in RUL space(top panel is for augmented data), while Table 5 summarizes the final cluster compositions. When trained with GAN-augmented data, components are grouped into fewer, more compact clusters with a silhouette score of 0.579, reflecting moderate but meaningful separation given the heterogeneous degradation profiles. In contrast, clustering based on the original (non-augmented) data yields more fragmented groupings, driven by sparse and uneven failure histories, resulting in a higher silhouette score (0.885) but reduced practical interpretability for coordinated maintenance actions. This higher silhouette score is primarily an artifact of exaggerated separation caused by sparse and unstable RUL estimates, rather than evidence of genuinely distinct degradation regimes. These results highlight the stabilizing affect of data augmentation on RUL-based clustering, enabling more coherent grouping of components for downstream maintenance scheduling.

**Table 5:** Clustering results.

Cluster	Components
*With Gans*	
0	{0, 4, 6,8}
1	{ 1, 2, 3}
2	{ 5,7}
*Silhouette score:*	*0.579*
*Original data*	
0	{0, 1, 2, 3, 4, 5, 6, 8, 9}
1	{ 13,15}
2	{ 12,14}
3	{10, 11}
*Silhouette score:*	*0.885*

#### Stage 2: Optimal maintenance time selection and sensitivity analysis

After clustering components based on RUL proximity, a grid search was conducted to determine the optimal maintenance time $t\_k$ for each cluster. The objective was to minimize the expected maintenance cost while avoiding both premature preventive actions and excessive failure risk. The search domain for ${t}_{k}$ ​ was defined over the feasible RUL range of each cluster, with the additional constraint that the normalized failure risk proxy remained below a predefined threshold.

Expected maintenance cost was expressed as a weighted combination of preventive, failure, and downtime costs. To enable a systematic sensitivity analysis, all costs were normalized by the preventive maintenance cost ${C}_{p}$ ​, such that ${C}_{p}$ = 1, while failure and downtime costs were expressed as multiples of ${C}_{p}$​ (i.e.,${C}_{pf}$ ∈ {5,10,20,40,80}, ${C}_{d}$ ∈ {0,2,5,10}). This normalization allows results to be interpreted independently of absolute monetary scales.

**Fig. 5:**
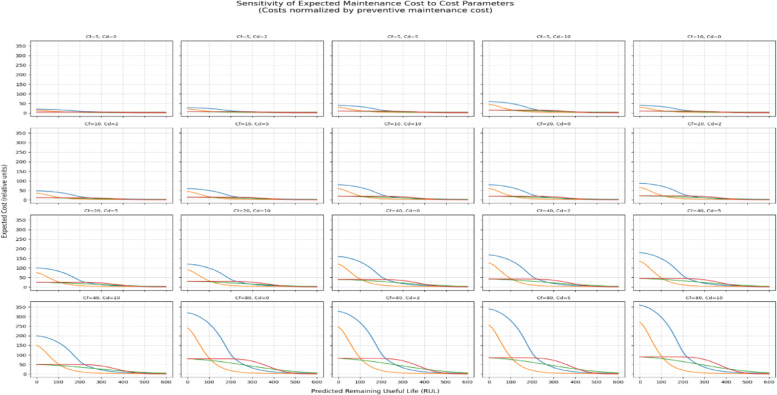
Trend in cluster costs (augmented data).

Figure 5 presents the sensitivity of the expected maintenance cost to variations in ${C}_{f}$ and ${C}_{d}$​ across the full RUL range. The results indicate that changes in cost parameters primarily rescale the magnitude of the expected cost curves without altering their overall shape. In particular, the location of the minimum expected cost—and thus the optimal maintenance time ${t}_{k}$​ remains stable across a wide range of cost assumptions. As expected, expected cost increases monotonically as ${t}_{k}$​ decreases toward zero RUL, reflecting the rapidly increasing risk of failure when maintenance is deferred excessively.

**Fig. 6:**
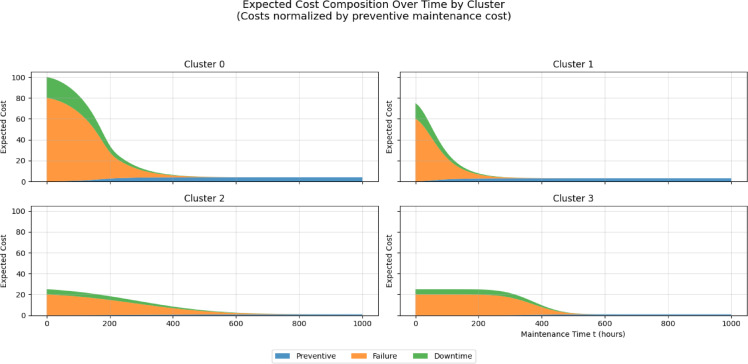
Decomposition of expected costs to preventive, failure and downtime costs (augmented data).

To further interpret cost behavior, Fig. 6 decomposes the expected cost into preventive, failure, and downtime components for each cluster. This decomposition confirms that variations in individual cost parameters uniformly magnify their respective contributions without changing the relative dominance or temporal ordering of cost components. Preventive cost dominates at large RUL values, while failure and downtime costs increase sharply as the system approaches the failure boundary.

**Fig. 7:**
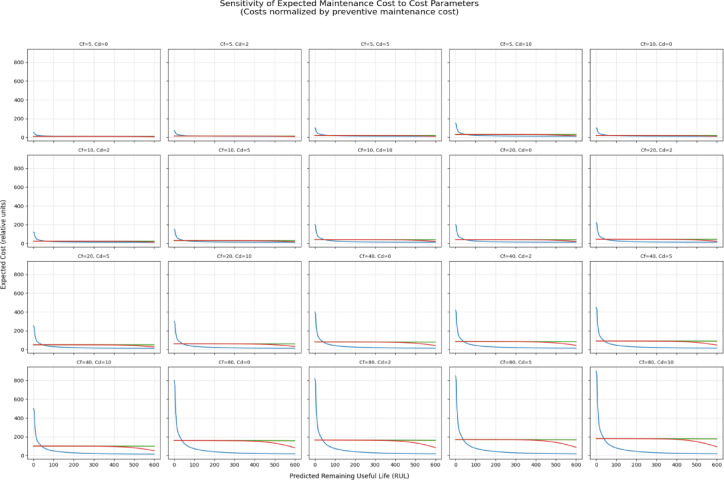
Trend in cluster costs(original data).

**Fig. 8:**
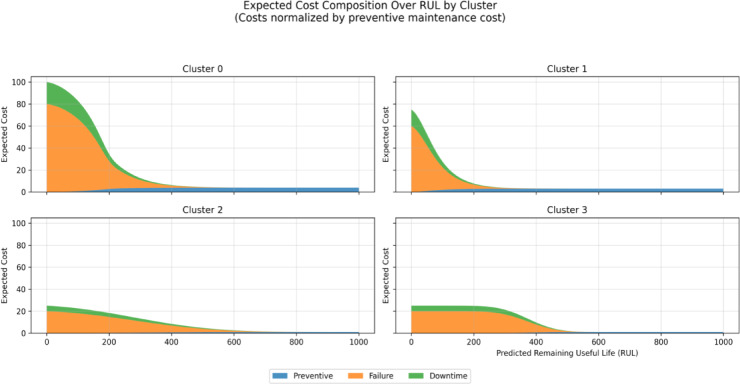
Decomposition of expected costs to preventive, failure and down time costs (original data).

An identical sensitivity analysis conducted on models trained without data augmentation produced qualitatively similar cost curve shapes and optimal maintenance times; however, the expected cost increased more steeply as RUL decreased, reflecting higher instability in risk estimates under data sparsity. See Figs. 7 and 8 below.

#### Stage 4: Validation-based comparison with baseline maintenance strategies

To further evaluate the effectiveness of the proposed cluster-based maintenance strategy, a validation-based comparison was conducted against two baseline scheduling approaches: a random maintenance strategy and fixed-percentile maintenance policies. All strategies were evaluated on the validation set using normalized cost parameters, consistent with the sensitivity analysis results.

For the proposed approach, maintenance scheduling was performed by combining RUL-based clustering with grid-search optimization of the maintenance threshold

${t}_{k}$​, subject to a minimum survival constraint (e.g., survival proxy ≥ 0.3). Maintenance actions occurring before failure was recorded as preventive, while actions scheduled after failure were counted as corrective interventions.

In order to further assess the efficiency of the proposed cluster-based maintenance strategy, a validation-based comparison was made on such two baseline scheduling strategies as random maintenance strategy and fixed-percentile maintenance policies. The entire strategies were tested on the validation set based on normalized cost parameters, which is in line with the sensitivity analysis outcome.

**Fig. 9:**
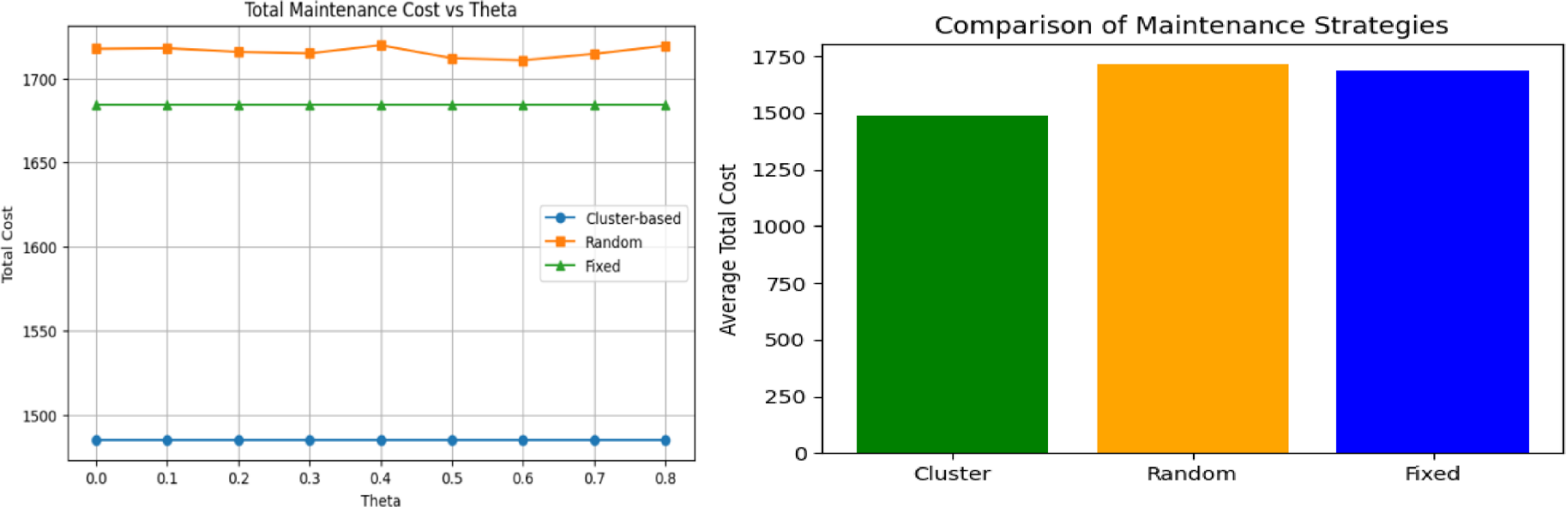
left panel shows trend in realized costs across survival threshold($\theta )$.while left panel shows the average costs(argumented data).

**Fig. 10:**
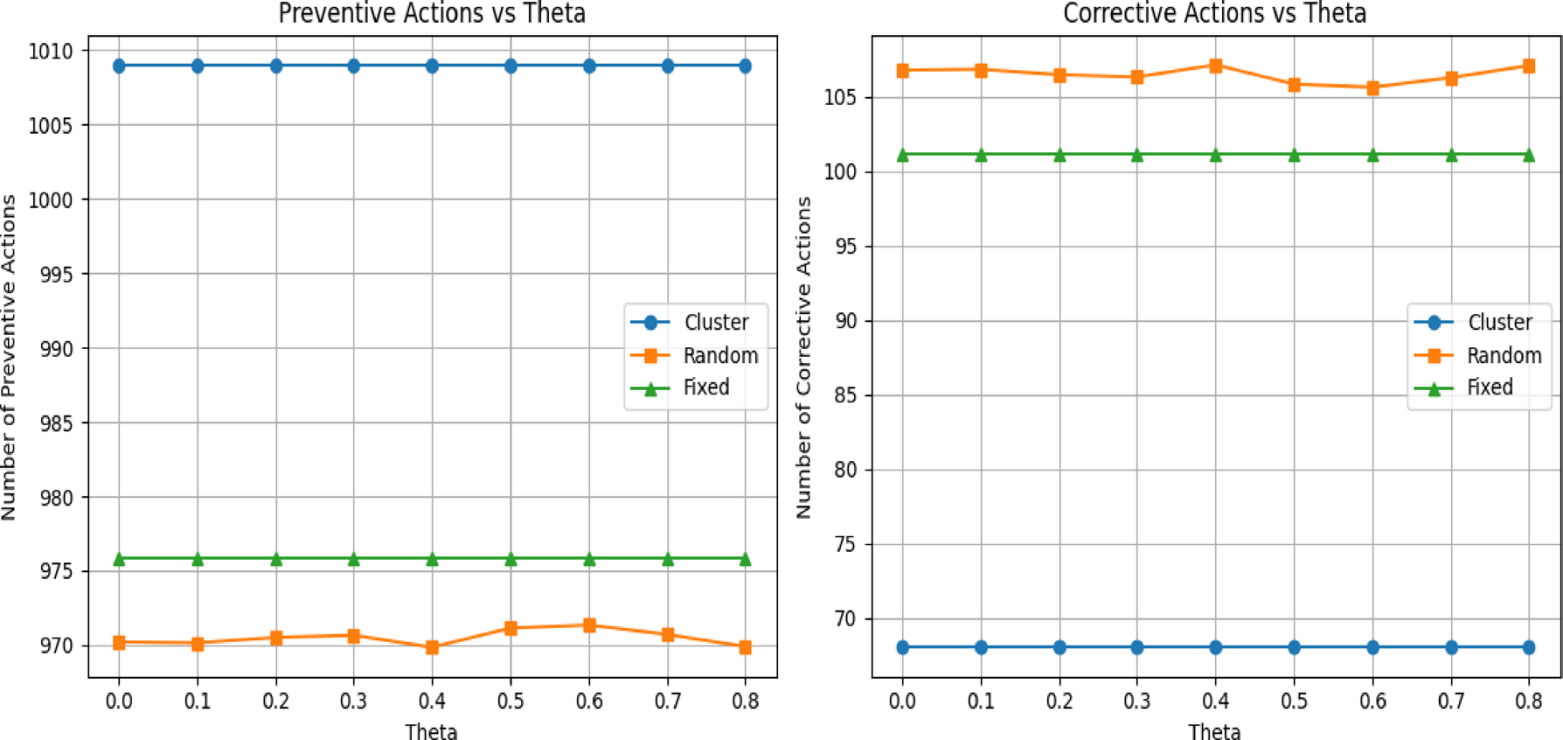
Comparison between predictive and correct maintenances (data augmented).

In the case of the proposed approach, maintenance scheduling was done by implementing a composite algorithm of RUL-based clustering and grid-search optimization of the maintenance threshold.${t}_{k}$, with a minimum survival constraint (e.g., survival proxy ≥ 0.3). The process of maintenance that takes place prior to failure was noted as preventive and the one executed after failure was noted as corrective interventions. Figure 9 below compares the three strategies based on Total costs and average of total costs while Fig. 10 compares the three strategies, based number of maintenance actions taken as $\theta$ changes.

Repeating the same evaluation using models trained without data augmentation yielded qualitatively similar trends, although the absence of augmentation resulted in higher corrective failure counts and increased cost variability. These results confirm that the proposed cluster-based strategy provides more reliable and cost-effective maintenance decisions than non-adaptive baseline policies, while remaining robust to cost scaling and modeling assumptions see appendix E for these graphs.

## Discussion

This study was motivated by three recurring limitations identified in the predictive maintenance (PdM) literature: (i) the treatment of data augmentation as a tool for improving predictive accuracy rather than decision robustness, (ii) the limited use of component-level clustering for coordinated maintenance actions, and (iii) the reliance on point RUL estimates for cost-based scheduling under uncertainty. The discussion below interprets the results explicitly in relation to these strands of prior work.

### RUL prediction errors and decision robustness

Several prior studies emphasize improving RUL prediction accuracy as the primary objective of PdM models^9,10,33,34^. Consistent with observations in these works, the present results show that percentage-based error metrics such as MAPE can become extremely large for small true RUL values, exceeding 300% for some components. Similar instability of relative error measures at low RUL has been noted implicitly in probabilistic prognostics studies, though its downstream implications are rarely discussed.

Importantly, the proposed framework does not rely on point RUL estimates for maintenance decisions. Instead, predicted RUL trajectories are aggregated and modeled probabilistically, and decisions are driven by relative degradation proximity and distribution-aware risk trends. This design directly addresses a limitation of prediction-centric PdM pipelines highlighted in, where errors in late-life predictions can propagate into unstable maintenance decisions. The findings indicate that although the accuracy of RUL against some components decreases, the clustering and scheduling processes are stable, which proves that the robustness of decisions can be separated without depending on the accuracy of point-prediction.

### Role of GAN-based augmentation beyond prediction accuracy

Existing GAN-based PdM studies are mainly tested on the augmentation benefits by measuring them with better predictive metrics. As an instance, Hakami et al.^12^ and similar studies^18–20^ prove that GANs are able to address data scarcity and imbalance in classes, contributing to an increase in model accuracy. These studies however conclude at the prediction phase and fail to investigate the impacts of augmentation on downstream maintenance choices.

The research findings of ablation demonstrate that the main advantage of GAN-based augmentation is not just the predictive accuracy, but the ability to stabilize learned RUL distributions in the case of extreme sparsity of failures. Unaugmented RUL distributions are also unstable, and tend to cluster fragments into fake clusters, and the expected cost curves have sharp gradients at the boundary of failure. Augmentation on the other hand generates more coherent clustering structures and smooth RUL distributions, facilitating the cost optimization. This directly fills the gap it has been established in the Introduction the lack of frameworks that explicitly relate augmented data to effective maintenance decision-making.

Although silhouette scores were worse with GAN-augmented clustering, this result indicates the decrease in artificial separation due to low and patchy histories of failures instead of reduced operational relevance. As with the findings of^30–32^ on maintenance clustering, the concept of operational coherence and coordinated servicing is more significant than cluster separation in geometry only.

### RUL-based clustering and opportunistic maintenance

In the maintenance setting, the concept of clustering has been applied in terms of task grouping and efficiency of planning^29–32^, yet it has not been utilized in the context of PdM, with most of the research being conducted on the system-level or isolated component-level prognostics^33–35^. Independent treatment of components may result in inefficient maintenance schedules in cases where there are several components with similar degradation horizons as pointed out by.

The findings of the clustering in this paper provide empirical evidence of the usefulness of component-level RUL-based structuring. The framework allows opportunistic maintenance by grouping components that have a similar state of degradation and minimize redundant interventions and synchronize servicing actions of components that tend to need maintenance in a similar time range. This strategy is more realistic with regard to the heterogeneous nature of industrial systems, which is in line with the arguments that have been presented in^34,35^.

### Cost sensitivity and robustness of scheduling decisions

Several studies of PdM are based on putative parameters of costs without investigating their impact on maintenance decisions^26,27,34^. To overcome this drawback, the current sensitivity analysis shows that different preventive, corrective, and downtime costs mainly re-scale the expected cost curves but do not change their shape and moves the optimal maintenance level. This was behavioral throughout cluster and augmentation conditions, and it implies that the overall scheduling choices are controlled by the degradation behavior and not arbitrary cost calibration.

It is interesting to note that the non-augmented models had steep gradients in the cost close to the failure boundary in that it was more unstable and susceptible to scheduling delays. This result supports the need to integrate augmentation, clustering, and probabilistic risk modeling to make sound choices in uncertainty.

### Comparison with baseline maintenance strategies

In line with previous criticisms of non-adaptive maintenance policies viewed as static^27,34^, the finding when compared to the random and fixed-percentile baselines indicates the weaknesses of non-adaptive scheduling policies. The variability of random scheduling was found to be high even with Monte Carlo averaging and fixed-percentile strategies were incapable of capturing component-specific degradation behavior.

Conversely, the proposed cluster-based approach steadily minimized the corrective failures and lower normalized cost was obtained on validation data. These findings are empirical evidence that successful PdM must involve coordination among the components and adaptive and risk-sensitive threshold selection as opposed to fixed or prediction-based policies.

## Conclusions

The given work illustrates that predictive maintenance systems should be shifted to more decision-oriented designs that are not focused on predictions. The proposed framework includes data augmentation with GANs, RUL prediction, component-centric clustering, and cost-conscience scheduling to overcome the major shortcomings that were observed in the current PdM literature. The findings indicate that strong maintenance decisions can be attained even when there is extreme data unavailability and uncertainty of prediction, which offers a viable avenue to real-life implementation.

### Limitations and future work

Although the framework is better robust with sparse failure data, extremely limited history components were unable to provide stable distribution fits and were executed in isolation. Such components could be better incorporated in future work by using hierarchical or transfer-learning methods. Also, the suggested CDF-based risk proxy is not a actual failure-time probability but a relative risk measure, which can be enhanced further by incorporating models of survival analysis; it should be emphasized that theoretical basis may be further bolstered. The generalization of the framework into the multi-system environment and dynamic cost structures is also a potential research direction in the future.

## Supplementary Information

Below is the link to the electronic supplementary material.

Supplementary Material 1

## Data Availability

The datasets used during the current study are available from the corresponding author on reasonable request.

## Code availability

The complete raw source code used for training and validation has been made publicly available at: https://github.com/AliH1409/Code.git
