# Supplementary material for: A two-stage framework for cost-sensitive predictive maintenance using deep learning, GANs, and risk-aware clustering
Source: Sci Rep. 2026 Mar 21;16:14442. doi: 10.1038/s41598-026-42910-4 (PMC13150005; doi:10.1038/s41598-026-42910-4)
Supplement: Supplementary file 1 — Supplementary Material 1 [file 41598_2026_42910_MOESM1_ESM.docx]

Supplementary File S1. Extended Figures and Results

This file contains additional results and visualizations referenced in the main manuscript, including:

- Full WGAN model training history plots for all machines (S1.1)
- Density-curve comparisons between real and synthetic failure data for all machines(S1.2)
- PCA comparison between real and synthetic data(S1.3)
- Full LSTM model training history plots for all machines (S1.4)
- List of all tried distributions (S1.5)
- Appendices to be annexed on the manuscript (S1.6)

S1.1 Full WGAN Model Training History

Includes training curves (critic loss, generator loss, and Wasserstein distance) for all 16 machines.

**Figure S1.1.1** – Machine 0 training history


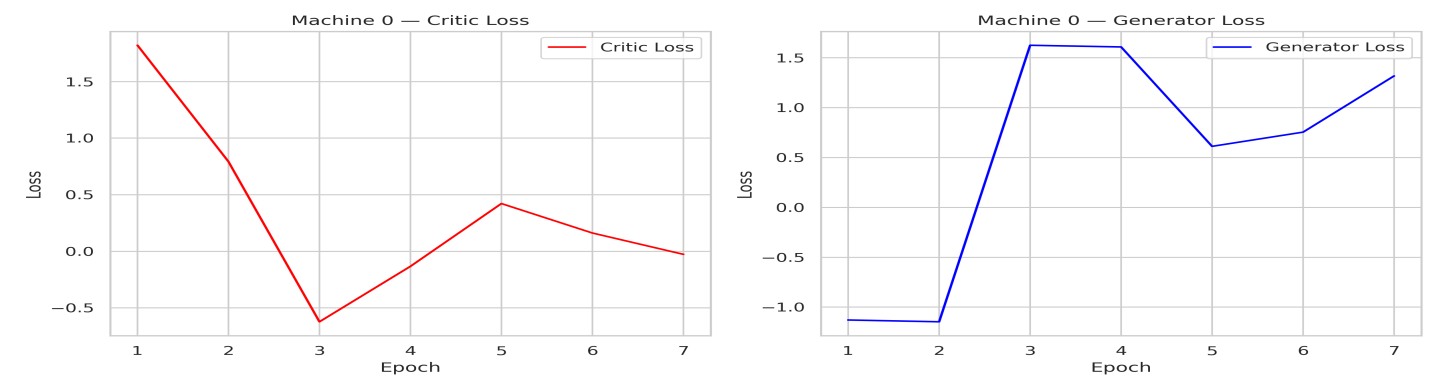


**Figure S1.1.2** – Machine 1 training history


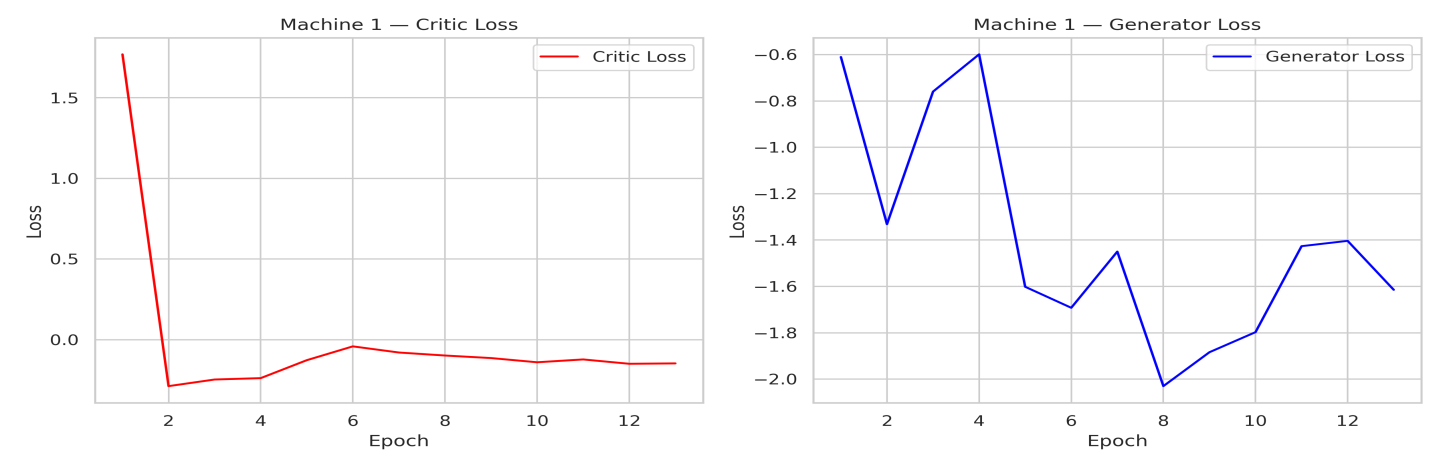


**Figure S1.1.3** – Machine 2 training history


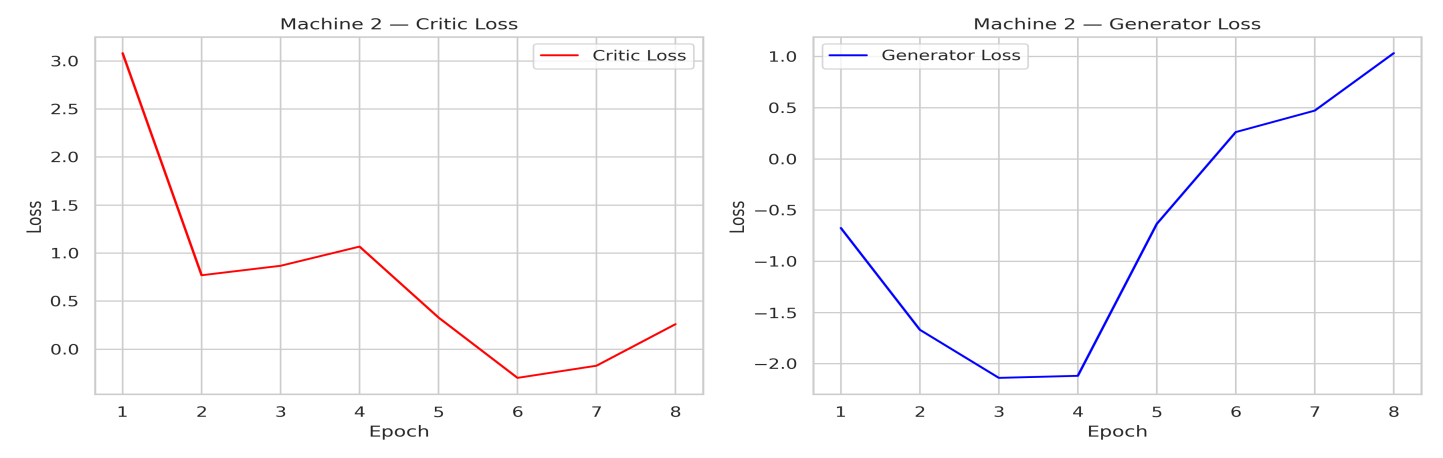


**Figure S1.1.4** – Machine 3 training history


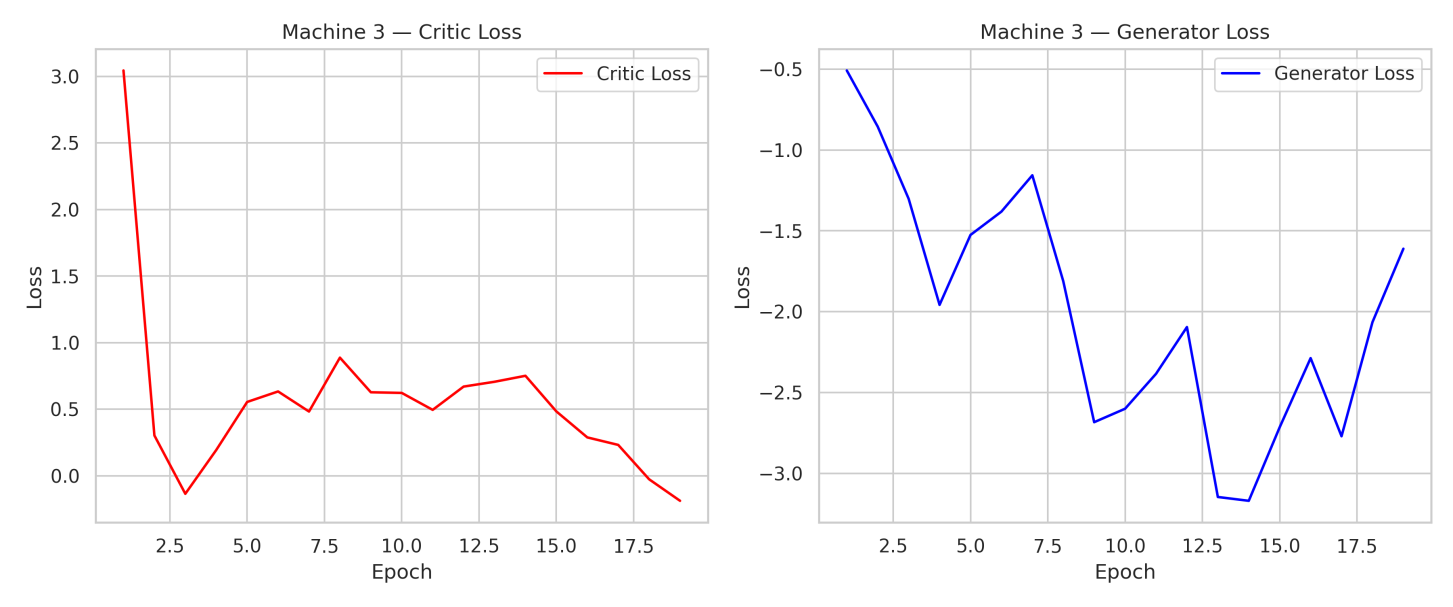


**Figure S1.1.5** – Machine 4 training history


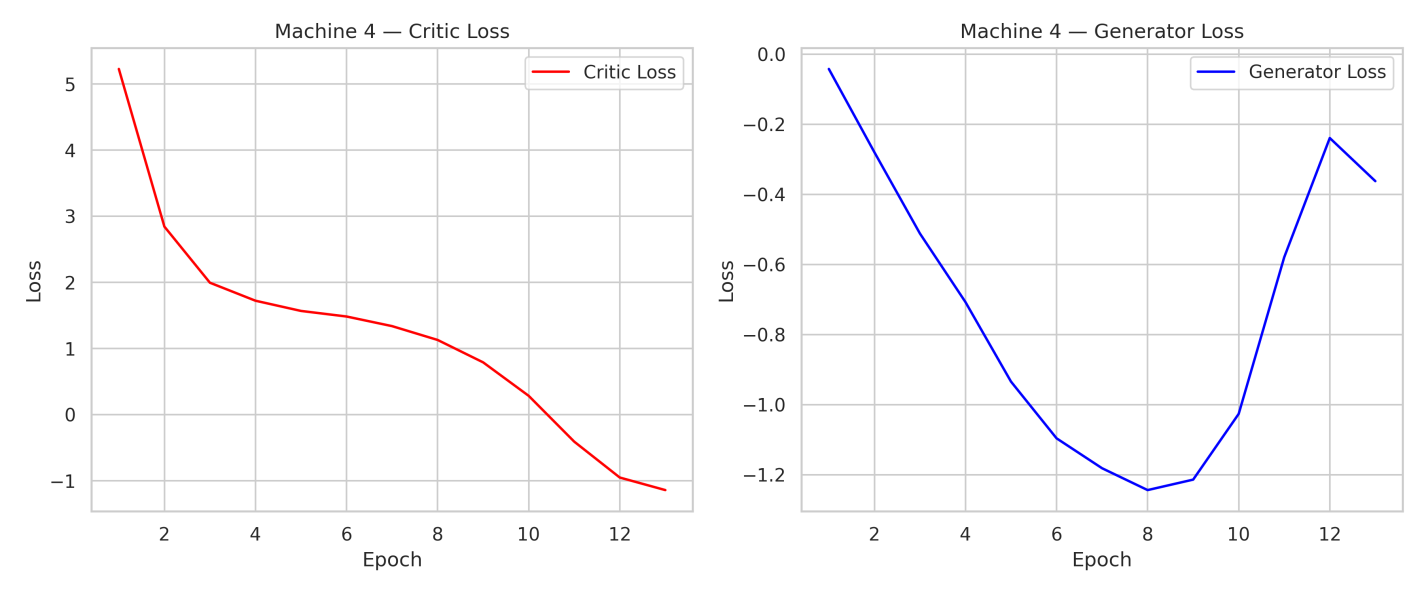


**Figure S1.1.6** – Machine 5 training history


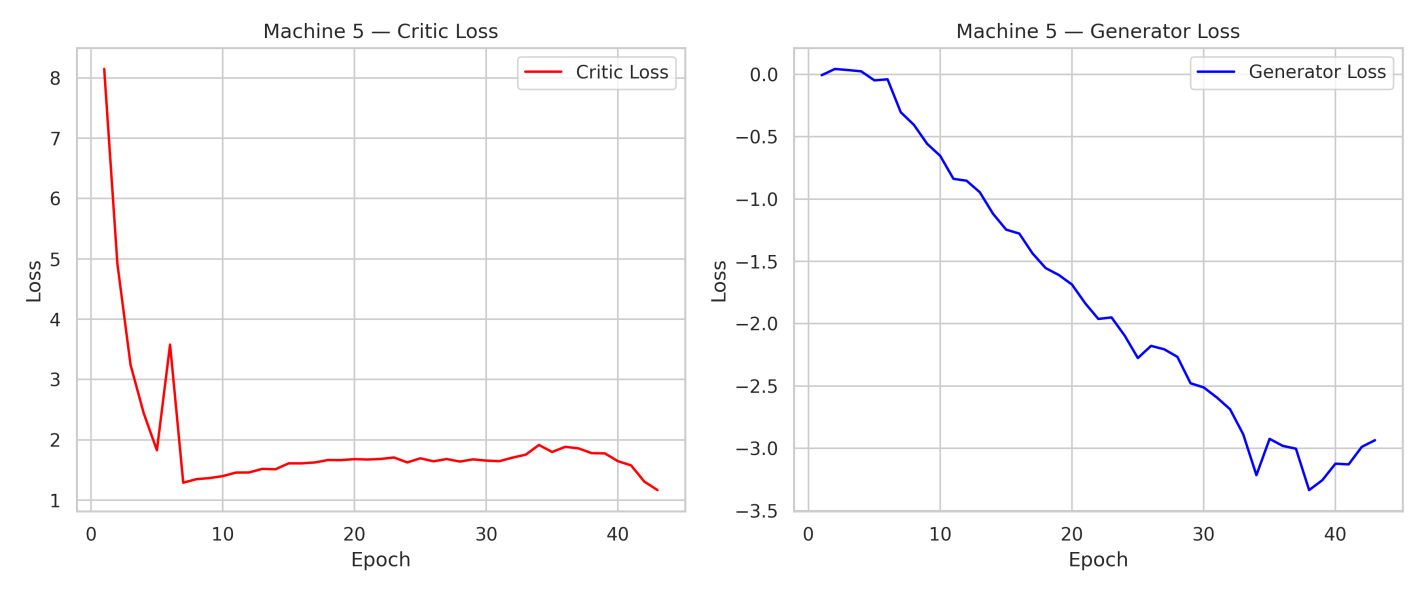


**Figure S1.1.7** – Machine 6 training history


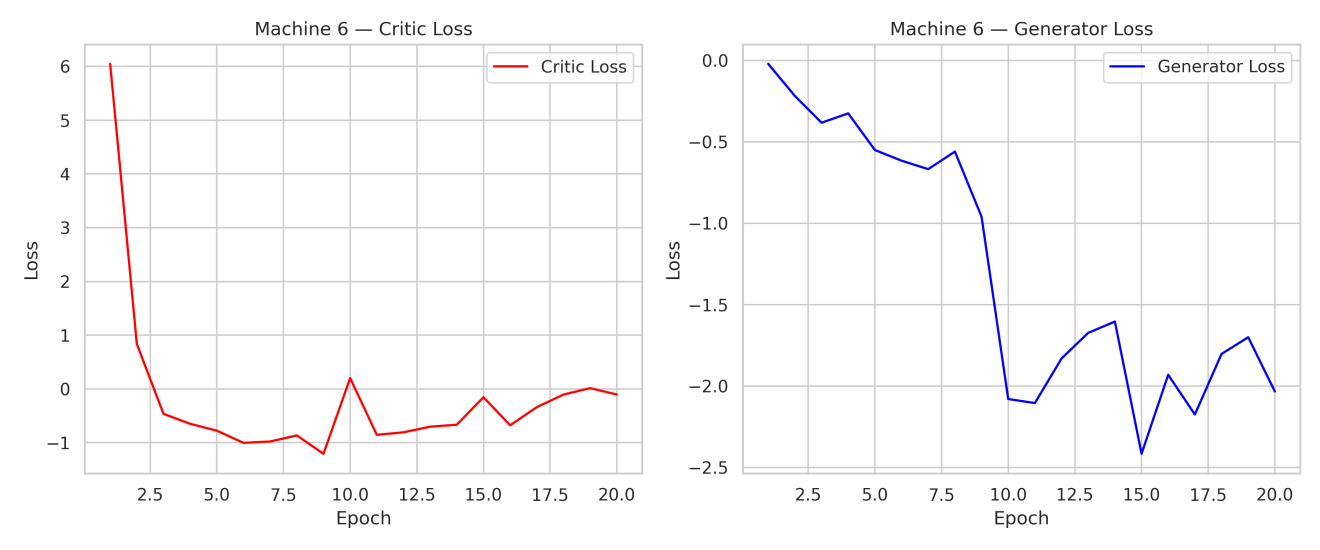


**Figure S1.1.8** – Machine 7 training history


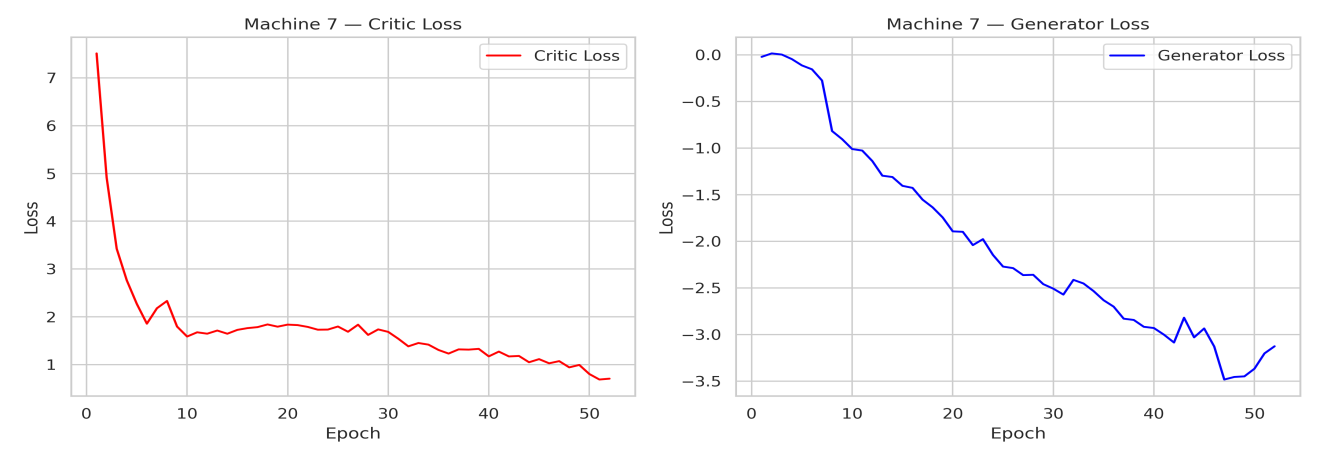


**Figure S1.1.9** – Machine 8 training history


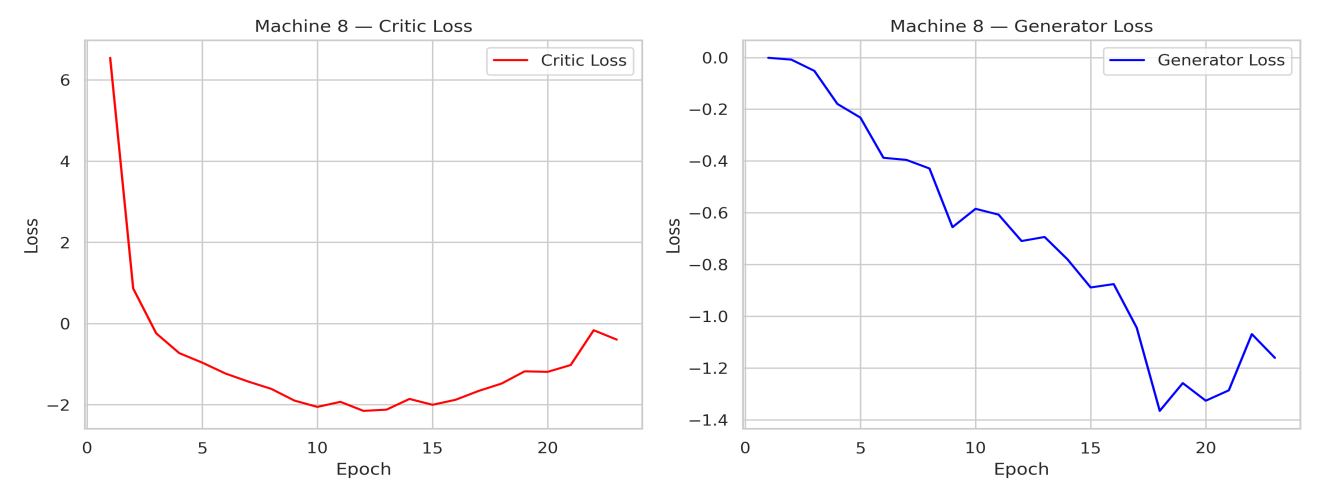


**Figure S1.1.10** – Machine 9 training history


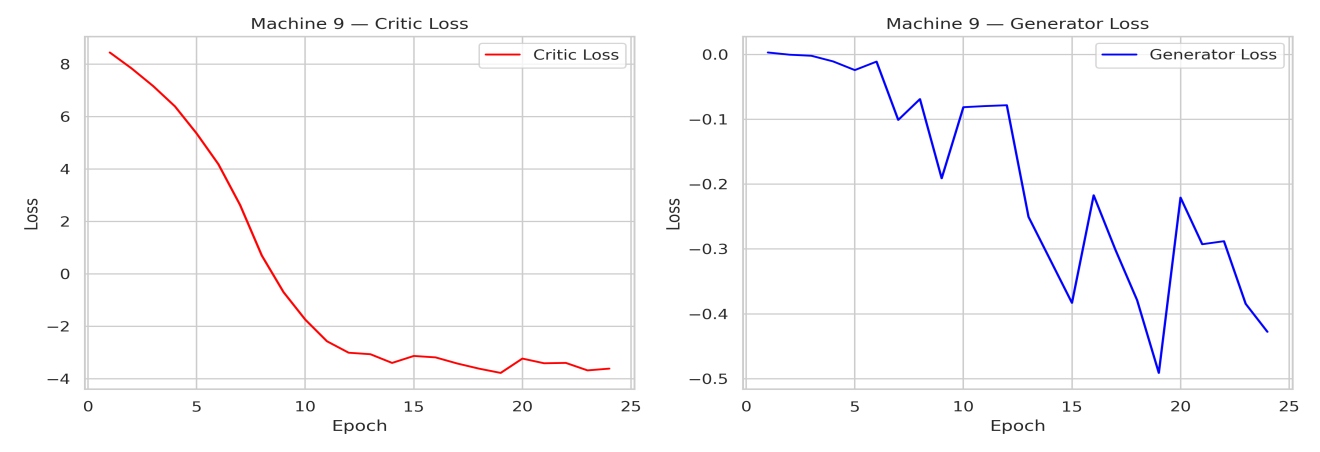


**Figure S1.1.11** – Machine 10 training history


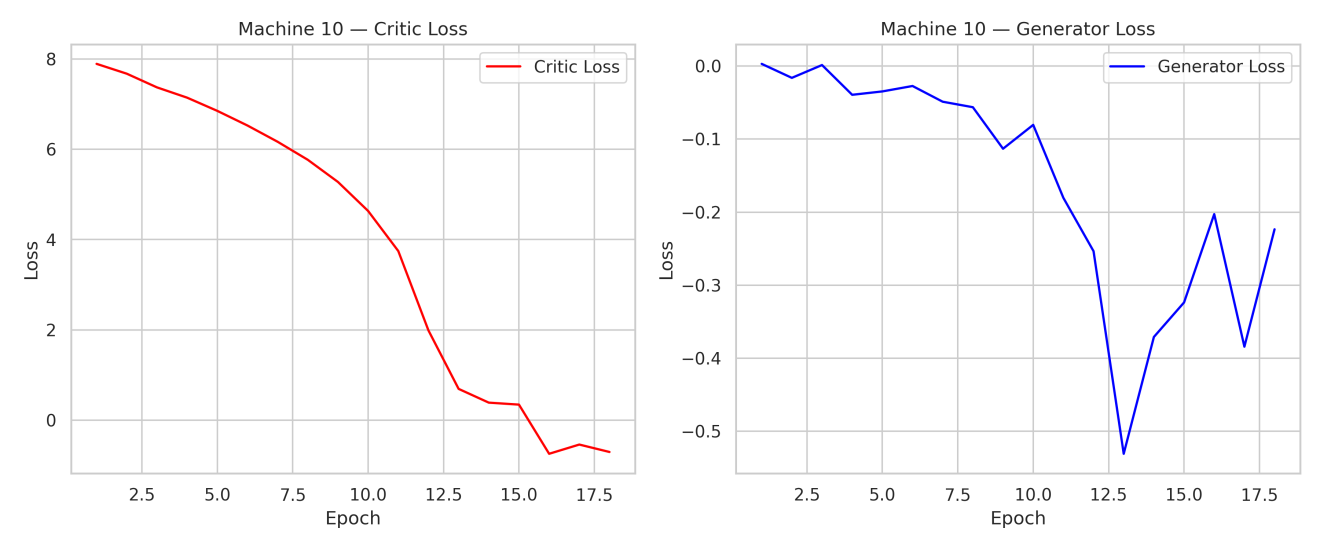


**Figure S1.1.12** – Machine 11 training history


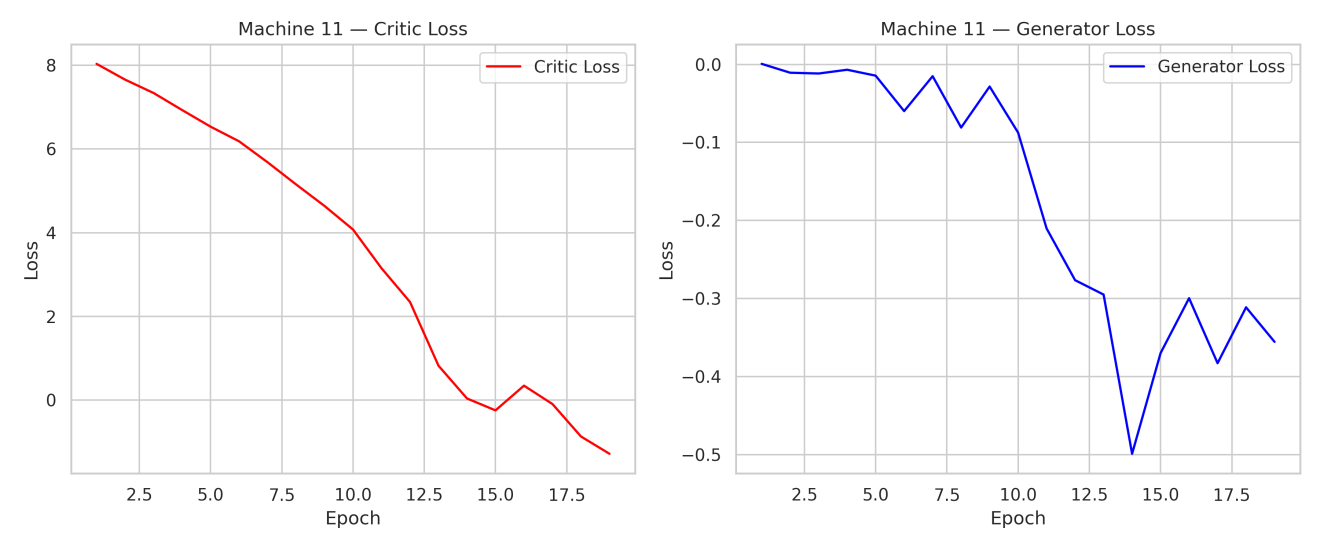


**Figure S1.1.13** – Machine 12 training history


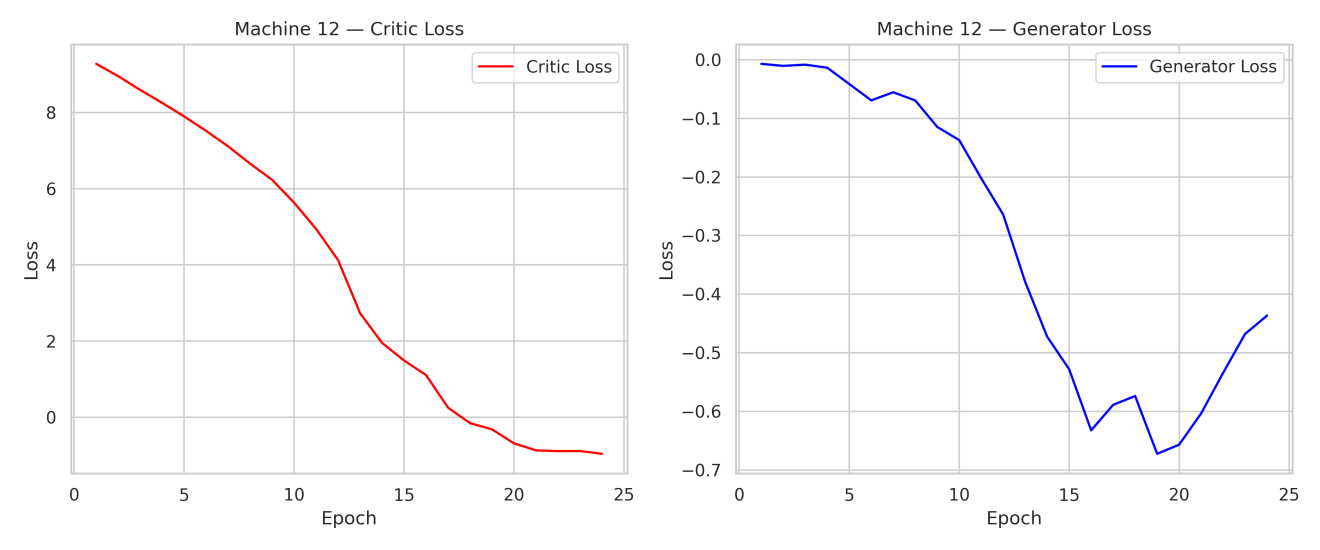


**Figure S1.1.14** – Machine 13 training history


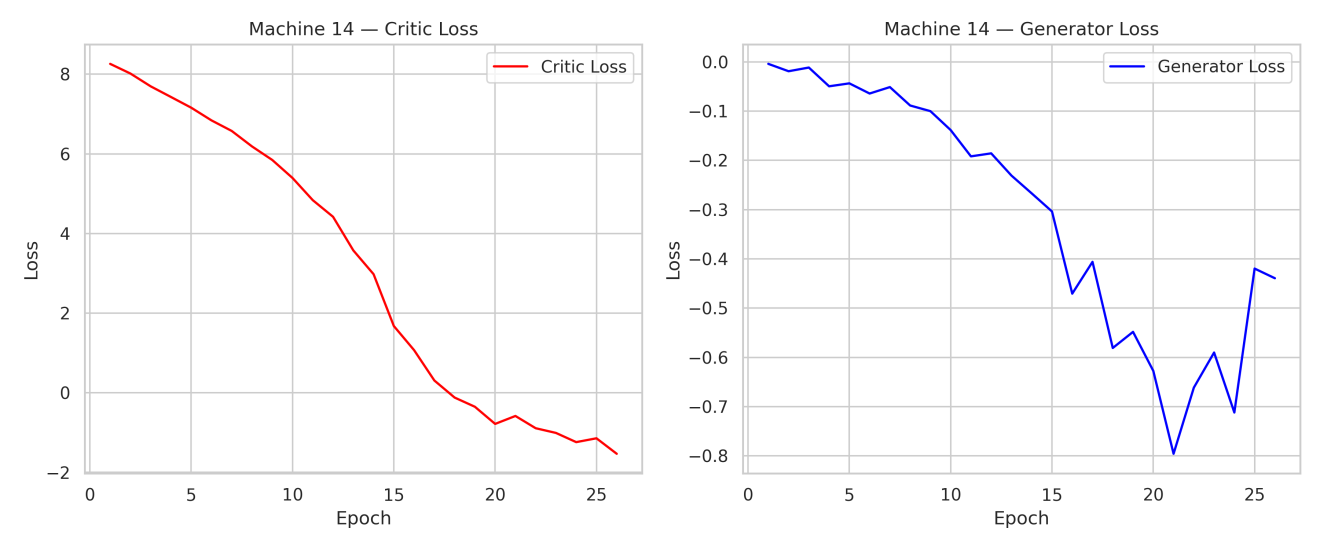


**Figure S1.1.15** – Machine 14 training history


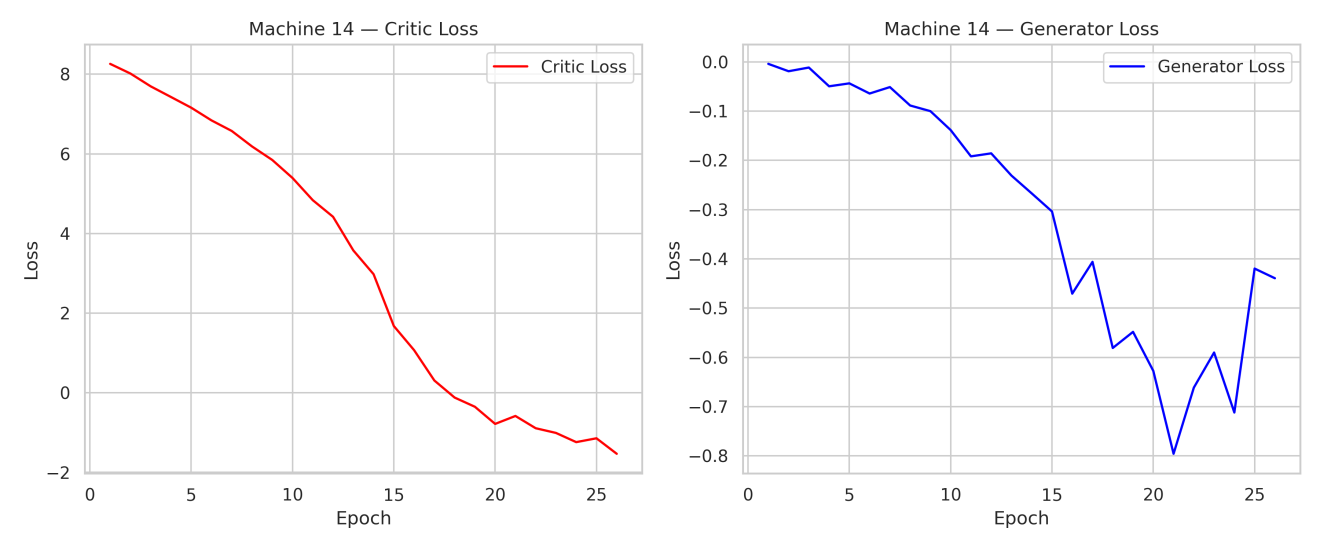


**Figure S1.1.16**– Machine 15 training history


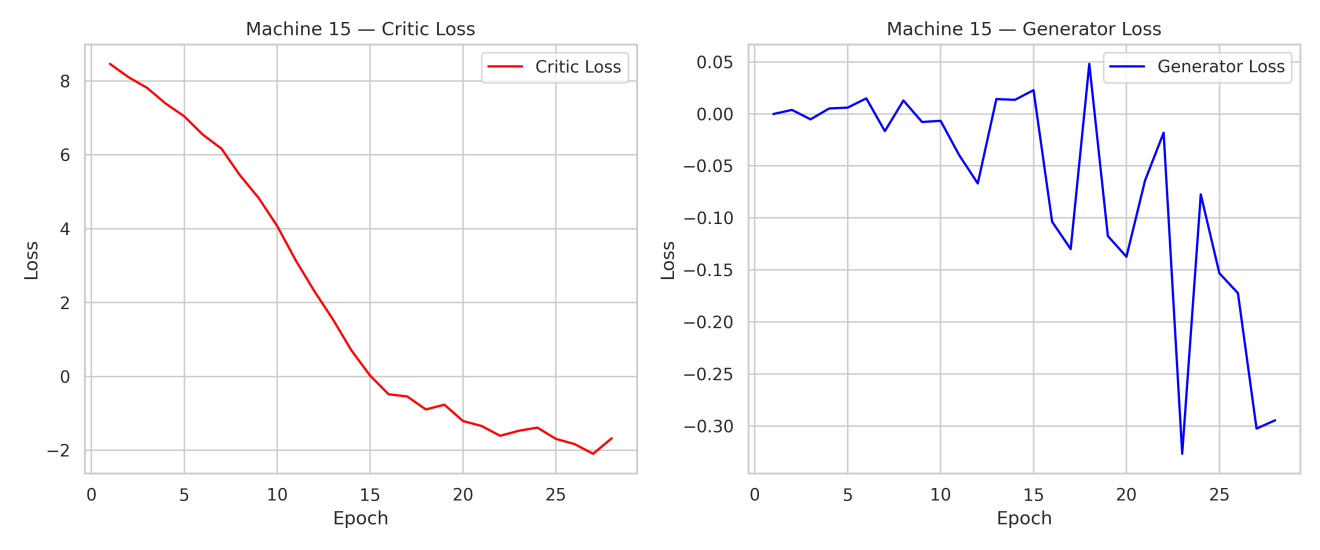


S1.2 Density-curve comparisons between real and synthetic failure data


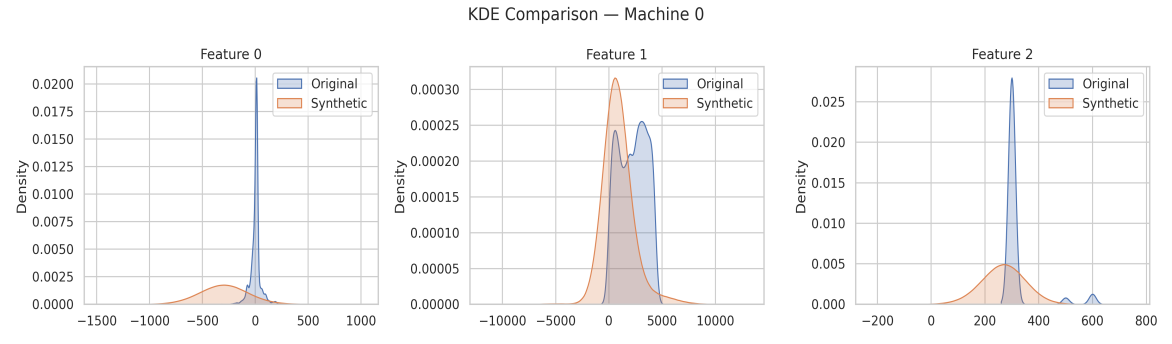


**Figure S1.2.1**– Machine 0 density curves


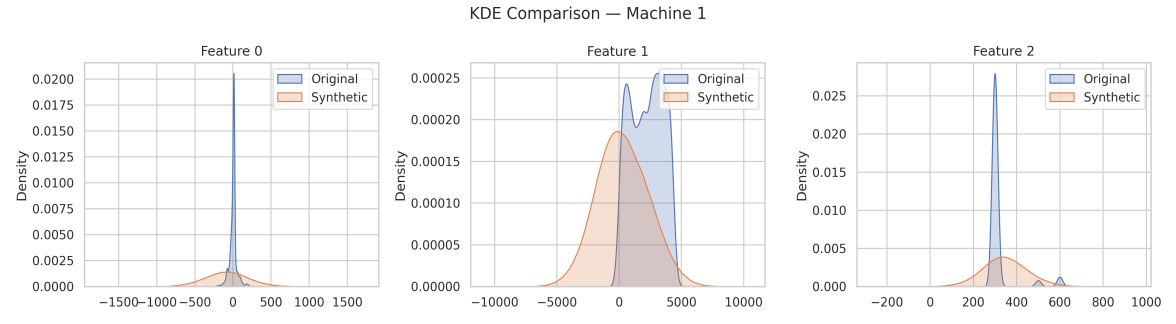


**Figure S1.2.2**– Machine 1 density curves


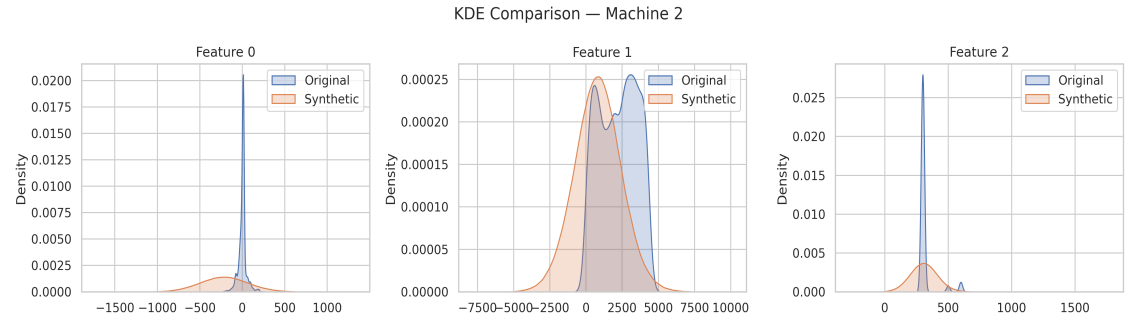


**Figure S1.2.3**– Machine 2 density curves


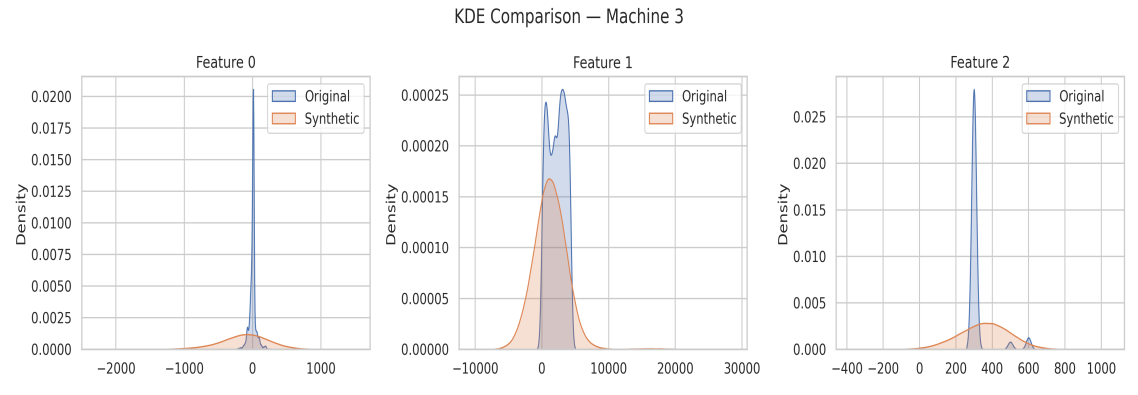


**Figure S1.2.4**– Machine 3 density curves


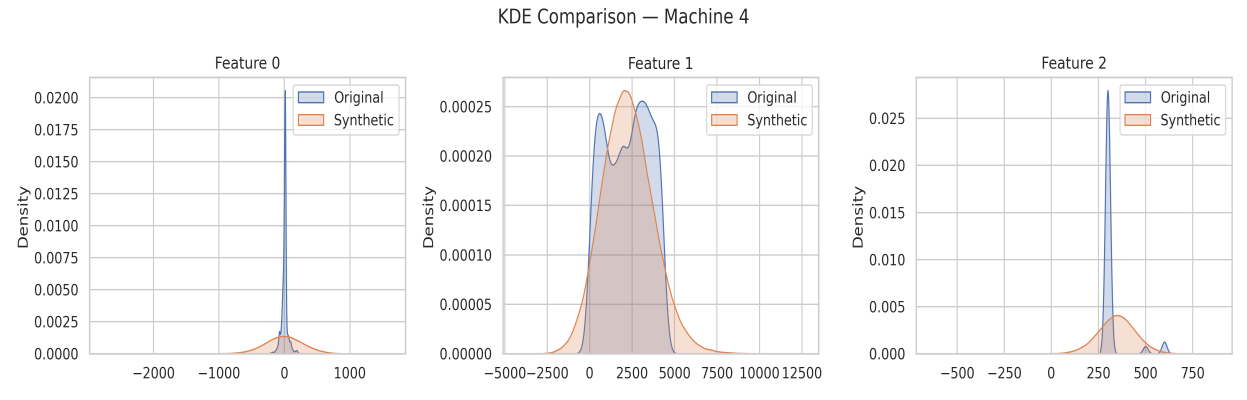


**Figure S1.2.5**– Machine 4 density curves


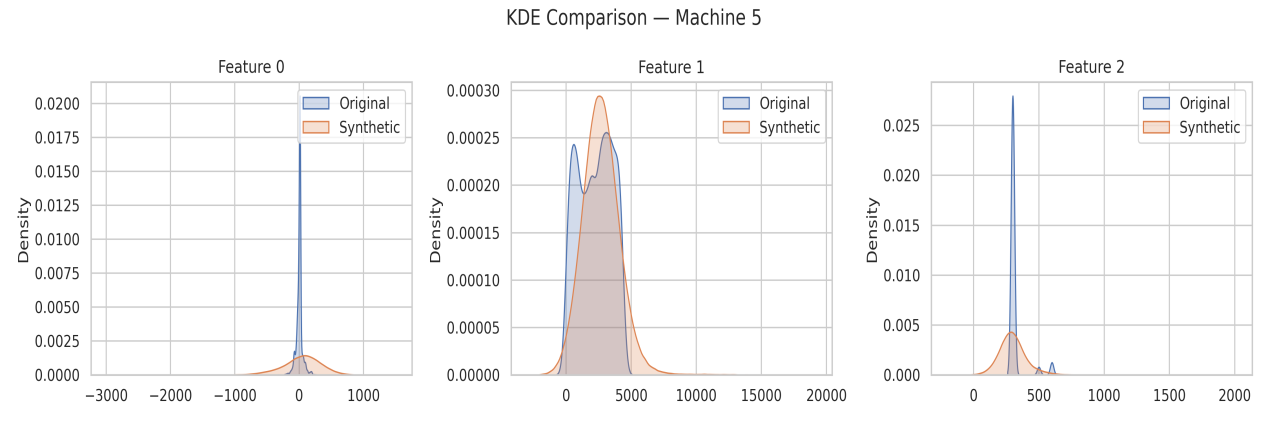


**Figure S1.2.6**– Machine 5 density curves


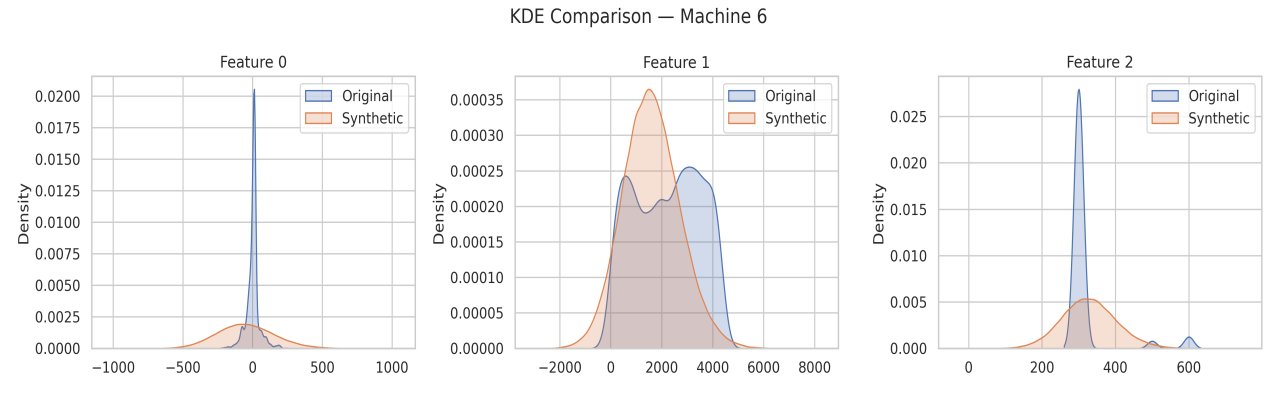


**Figure S1.2.7**– Machine 6 density curves


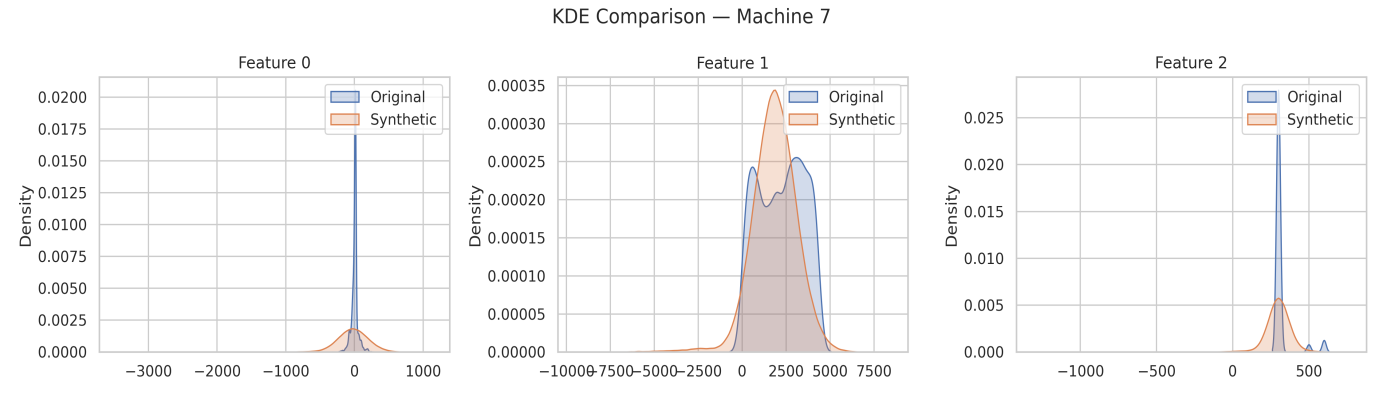


**Figure S1.2.8**– Machine 7 density curves


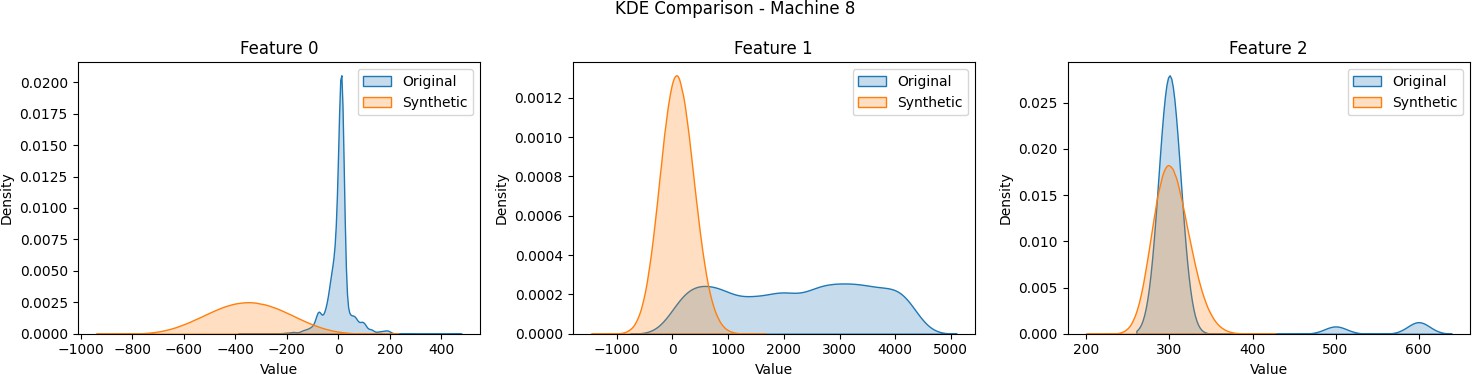


**Figure S1.2.9**– Machine 8 density curves


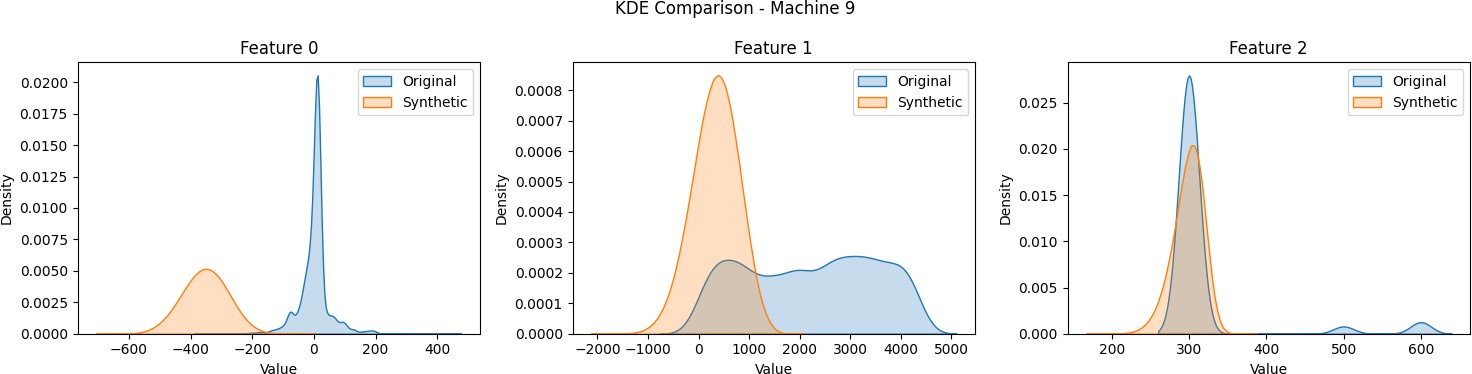


**Figure S1.2.10**– Machine 9 density curves


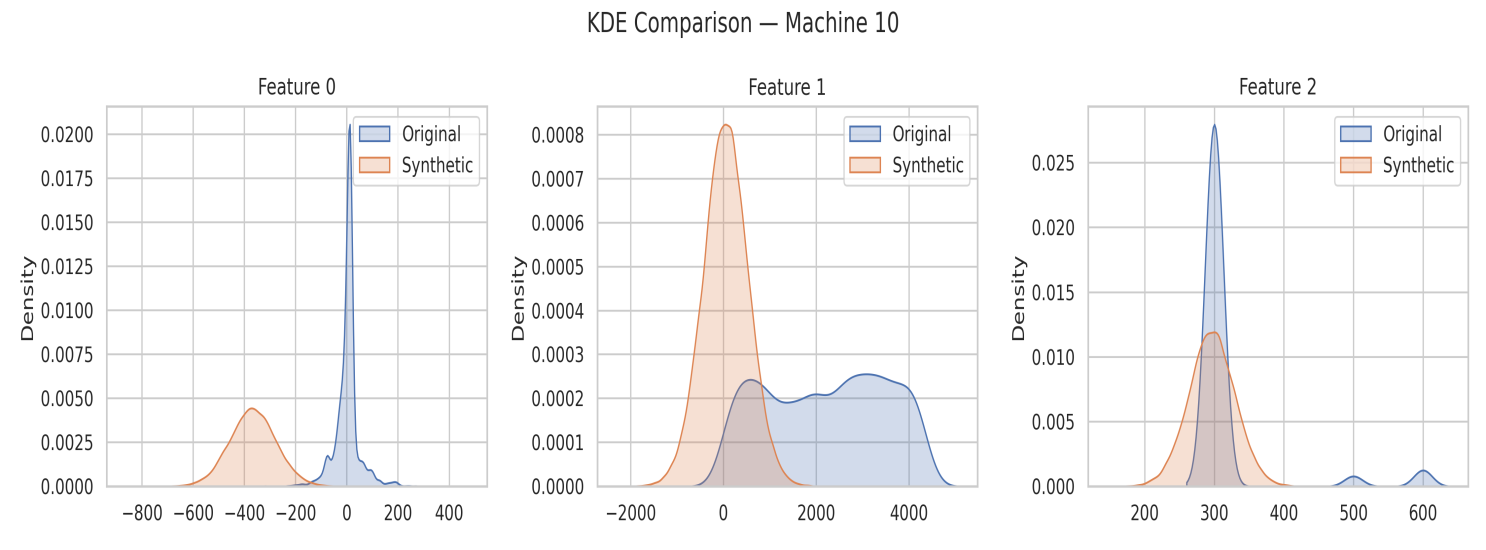


**Figure S1.2.11**– Machine 10 density curves


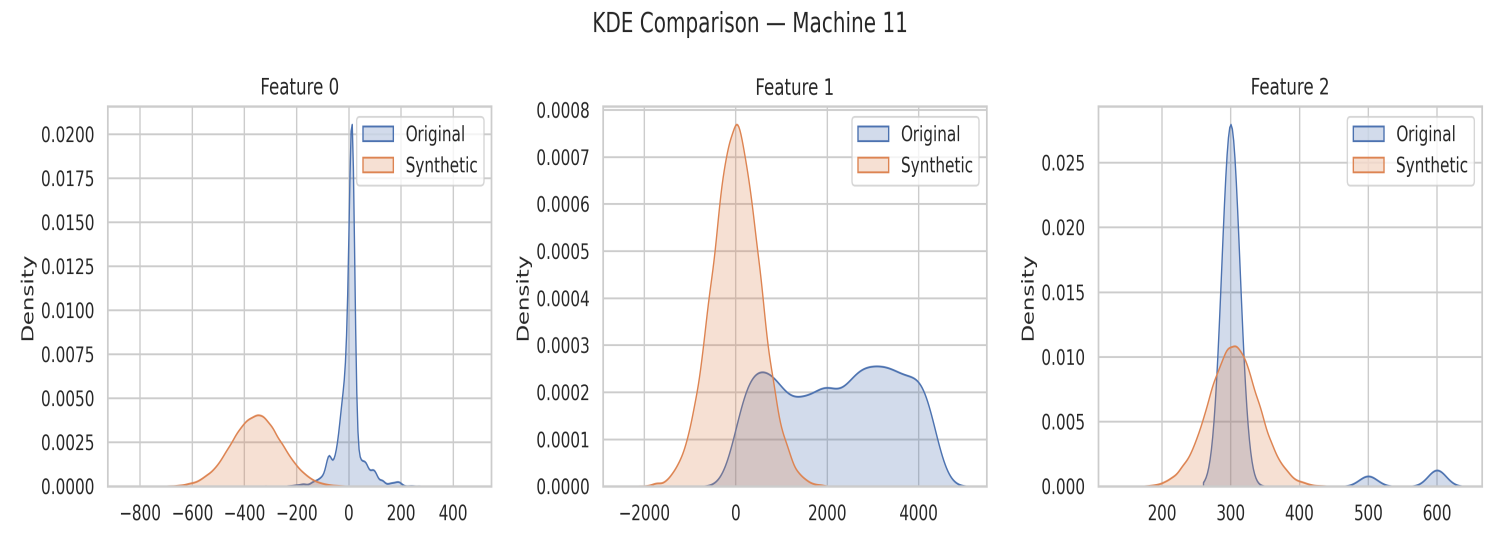


**Figure S1.2.12**– Machine 11 density curves


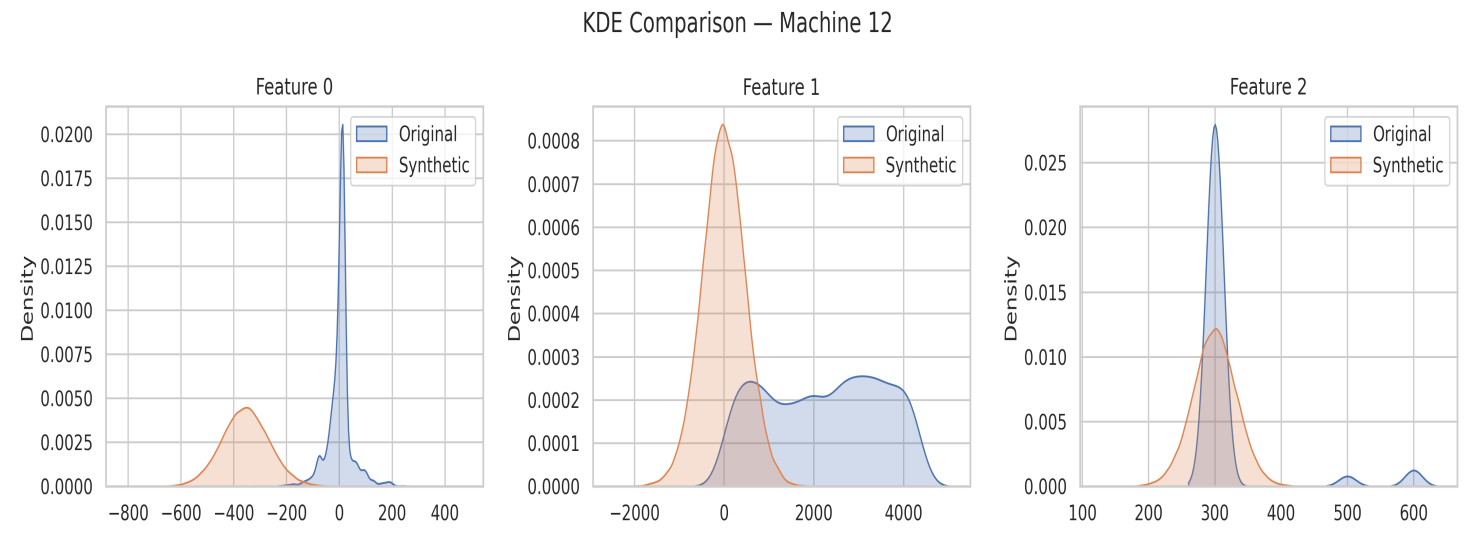


**Figure S1.2.13**– Machine 12 density curves


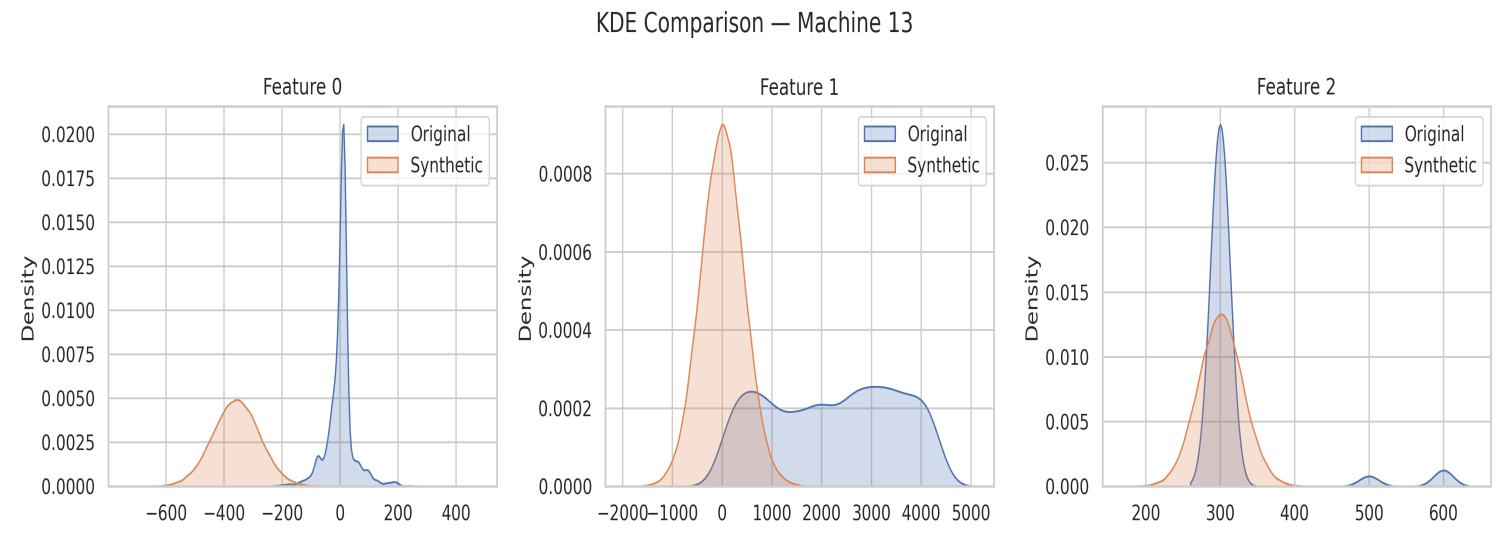


**Figure S1.2.14**– Machine 13 density curves


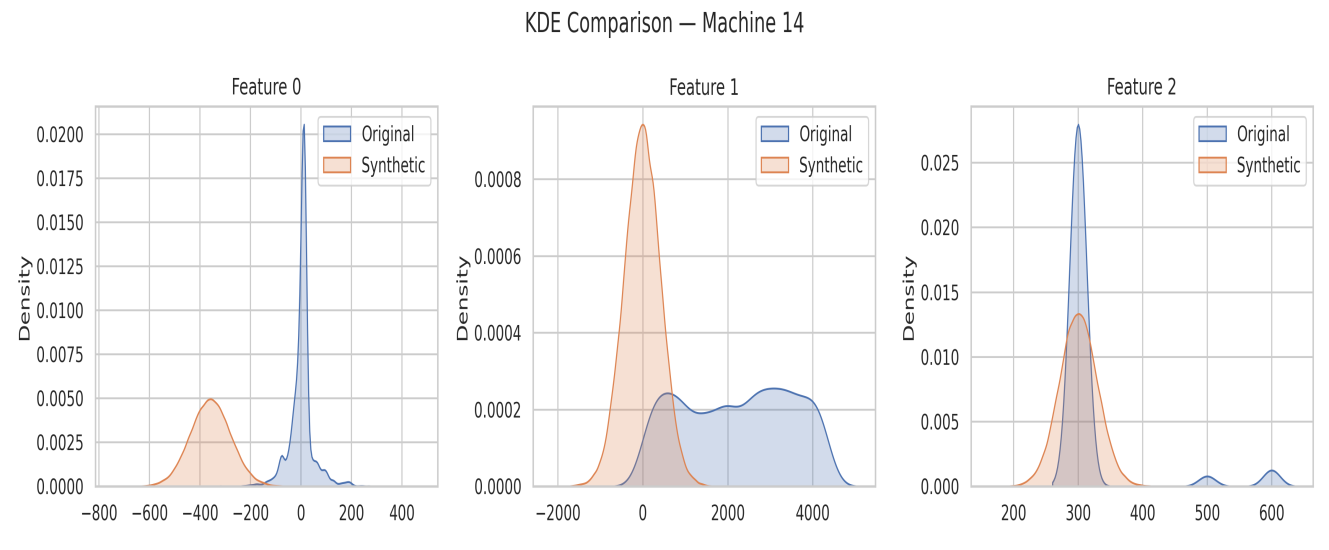


**Figure S1.2.15**– Machine 14 density curves


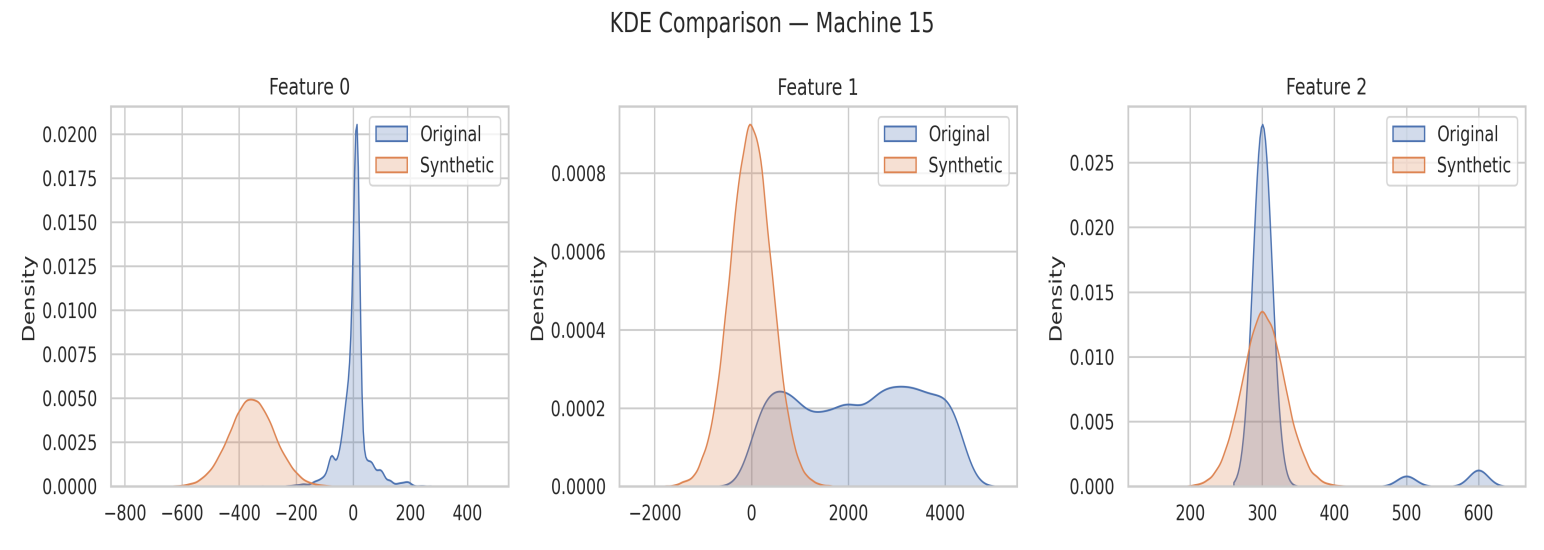


S1.3 PCA-Based Comparisons Between Real and Synthetic Data


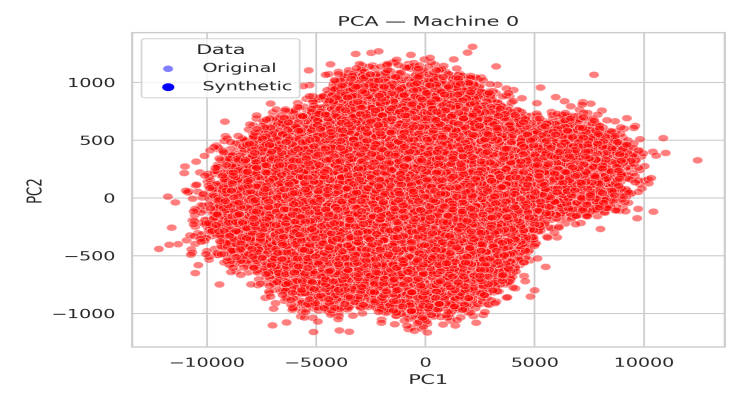


S1.3.1 PCA comparison between real and synthetic data for Machine 0


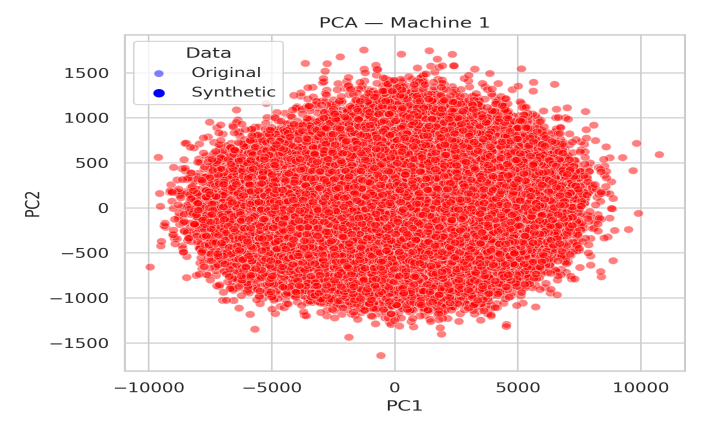


S1.3.2 PCA comparison between real and synthetic data for Machine 1


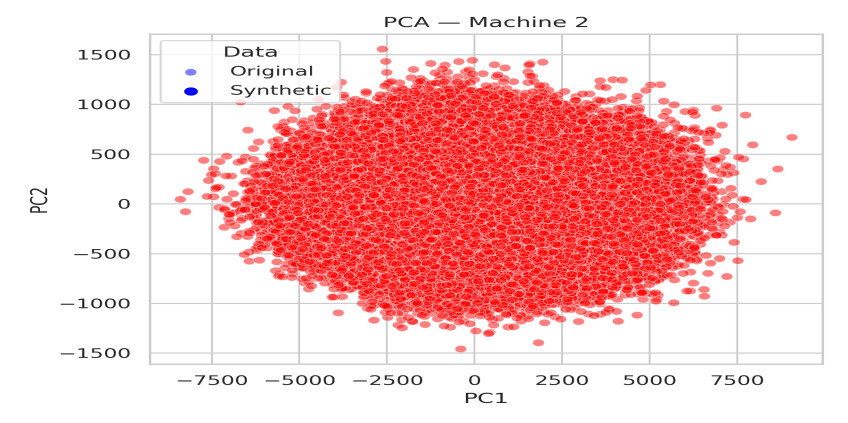


S1.3.3 PCA comparison between real and synthetic data for Machine 2


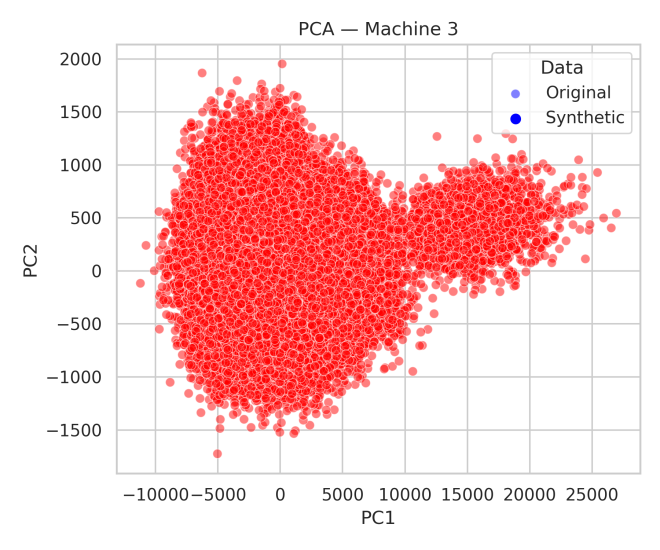


S1.3.4 PCA comparison between real and synthetic data for Machine 3


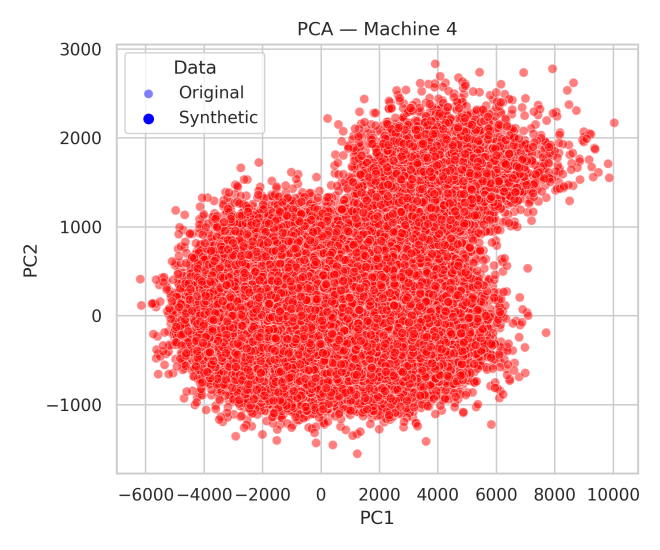


S1.3.5 PCA comparison between real and synthetic data for Machine 4


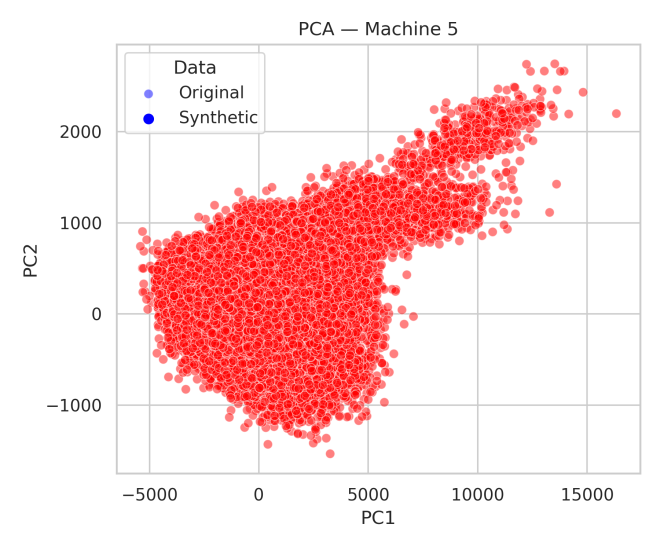


S1.3.6 PCA comparison between real and synthetic data for Machine 5


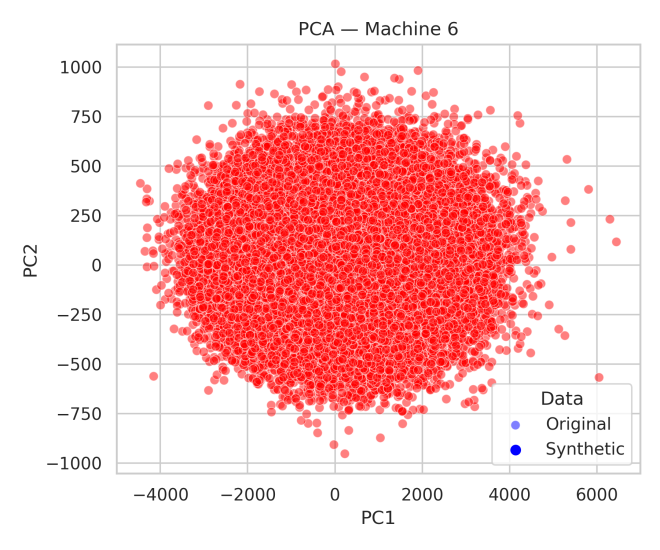


S1.3.7 PCA comparison between real and synthetic data for Machine 6


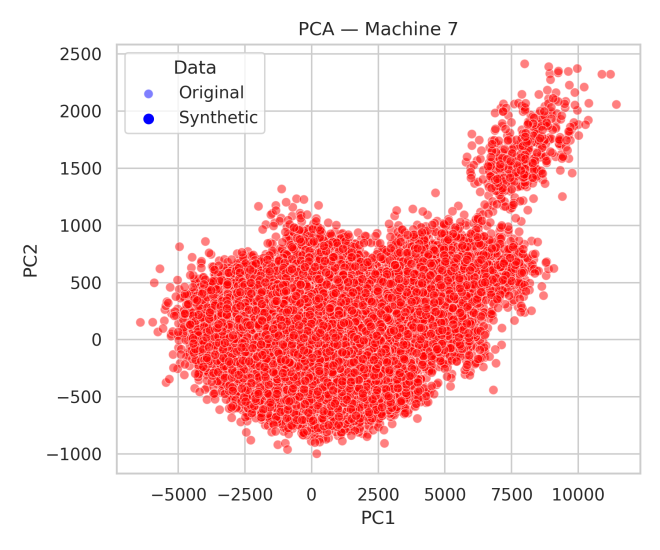


S1.3.8 PCA comparison between real and synthetic data for Machine 7


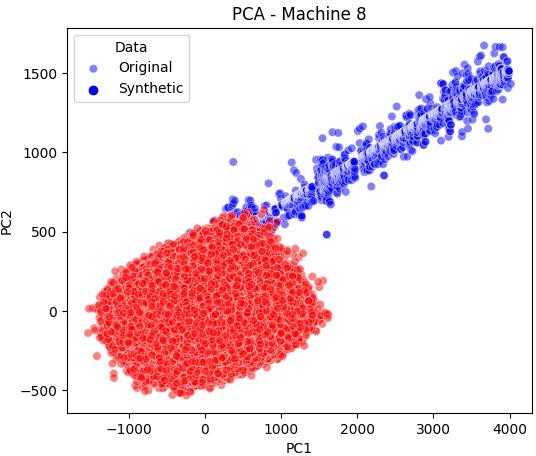


S1.3.9 PCA comparison between real and synthetic data for Machine 8


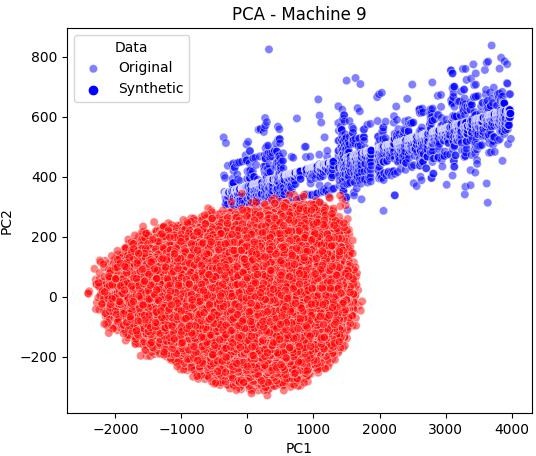


S1.3.10 PCA comparison between real and synthetic data for Machine 9


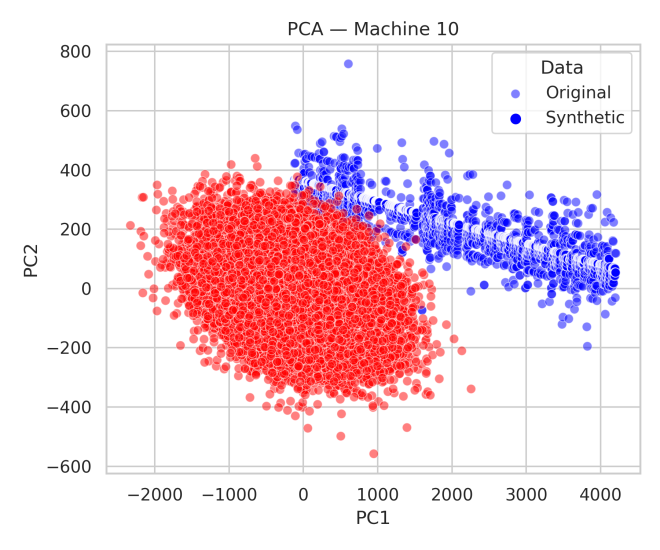


S1.3.11 PCA comparison between real and synthetic data for Machine 10


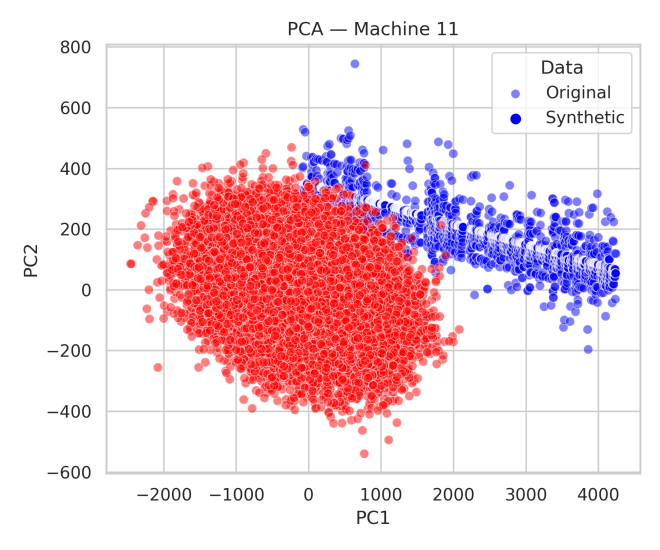


S1.3.12 PCA comparison between real and synthetic data for Machine 11


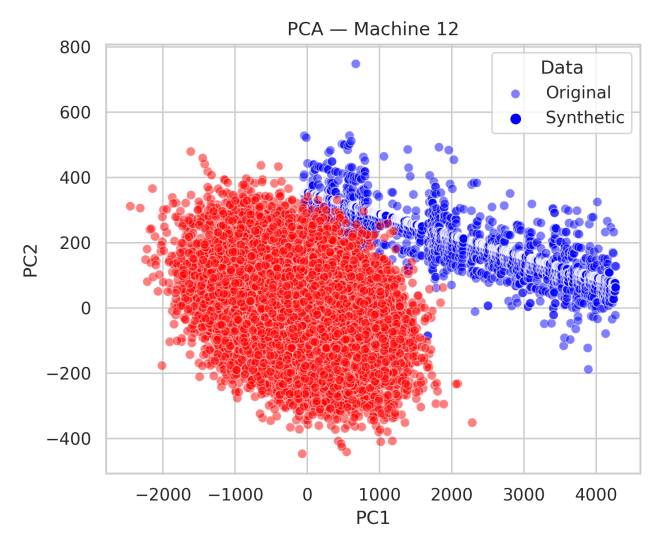


S1.3.13 PCA comparison between real and synthetic data for Machine 12


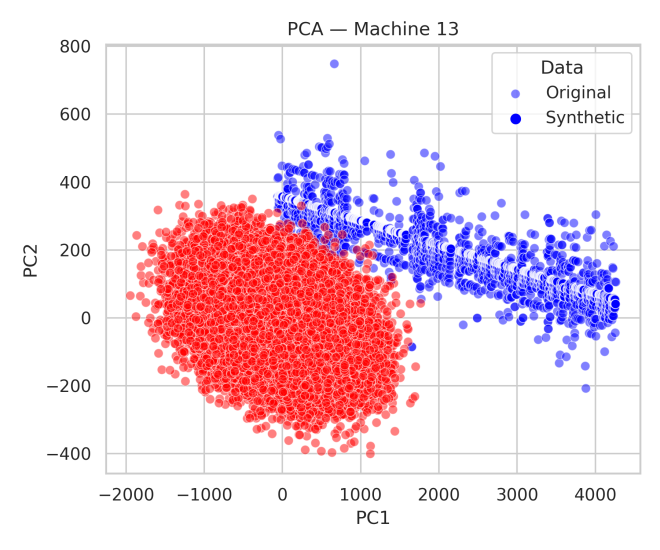


S1.3.14 PCA comparison between real and synthetic data for Machine 13


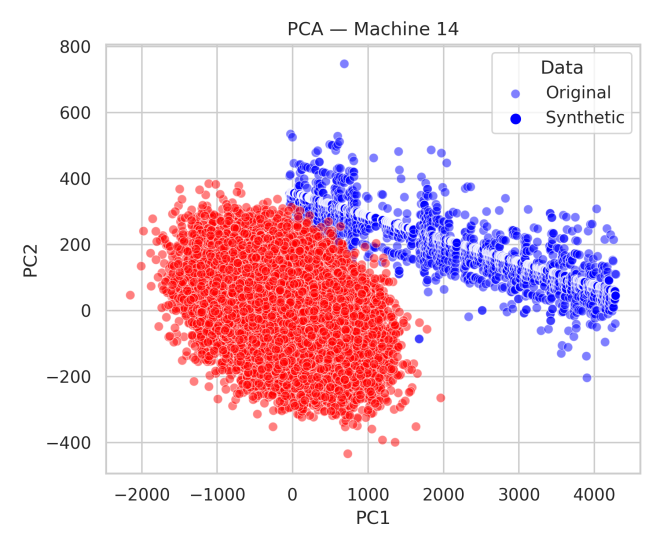


S1.3.15 PCA comparison between real and synthetic data for Machine 14


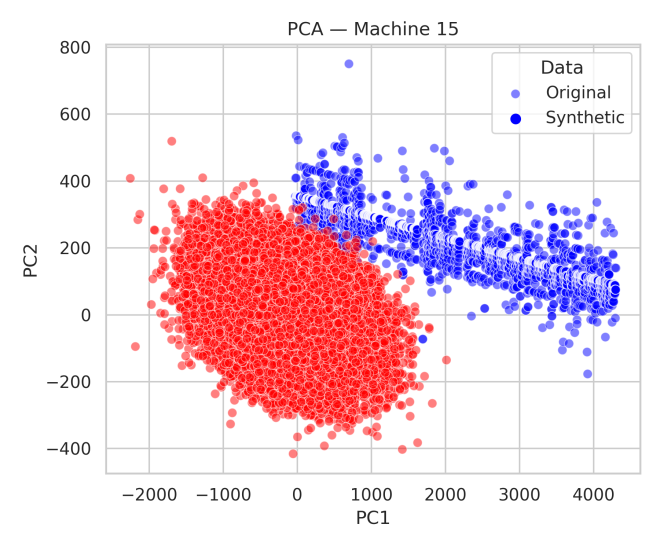


S1.3.16 PCA comparison between real and synthetic data for Machine 15

S1.4 Full LSTM Model Training History


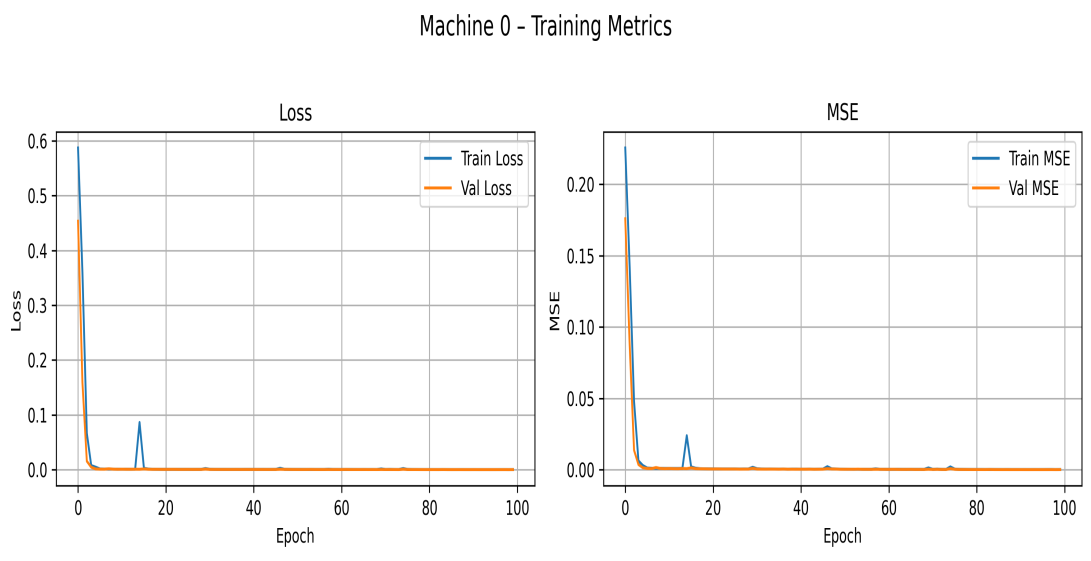


**S1.4.1** – Machine 0 LSTM training history


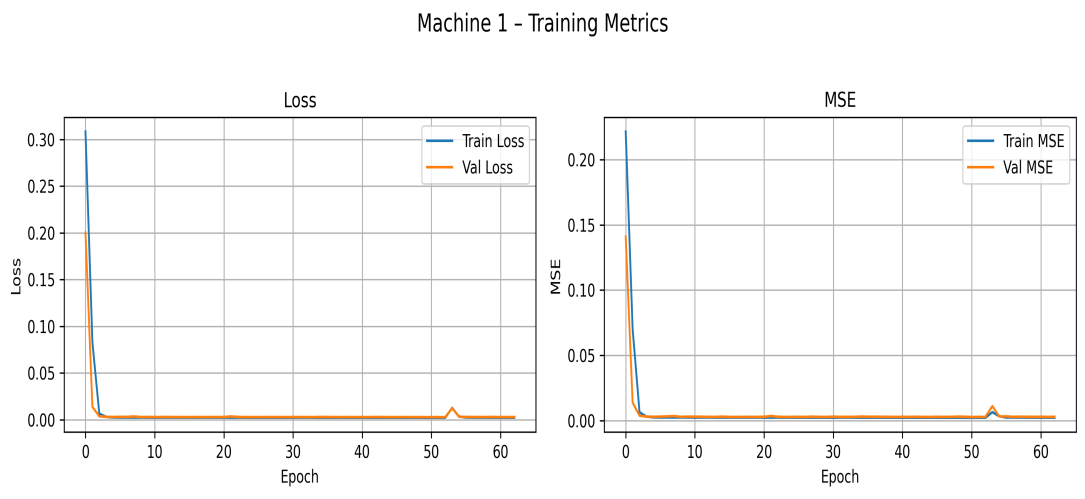


**S1.4.2** – Machine 1 LSTM training history


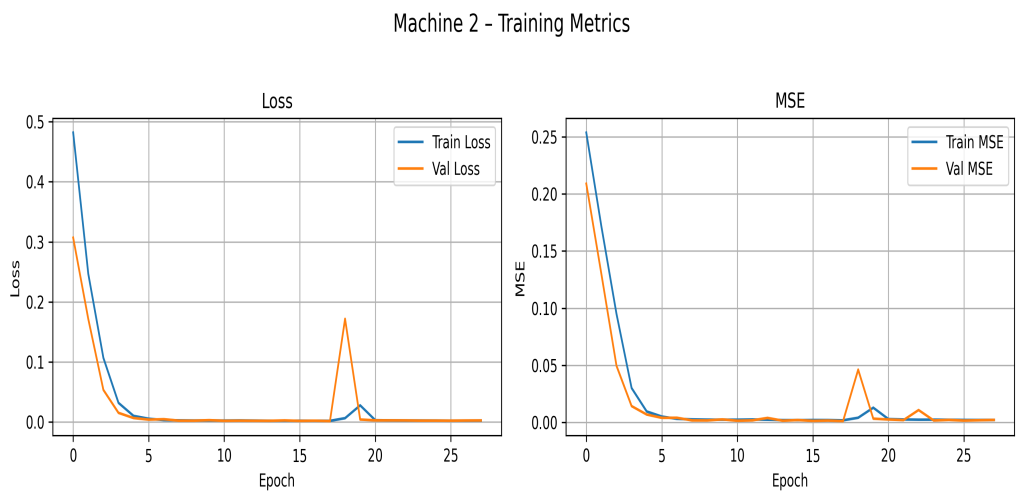


**S1.4.3** – Machine 2 LSTM training history


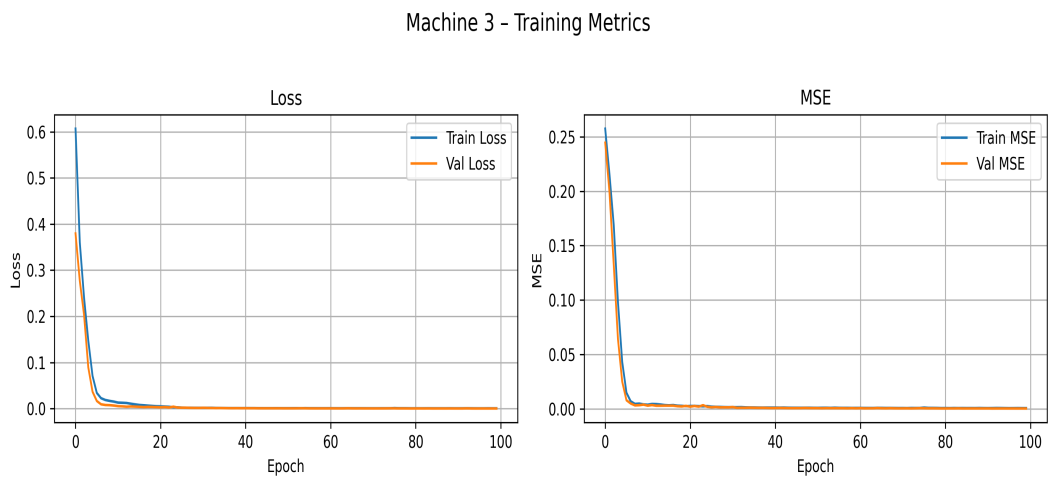


**S1.4.4** – Machine 3 LSTM training history


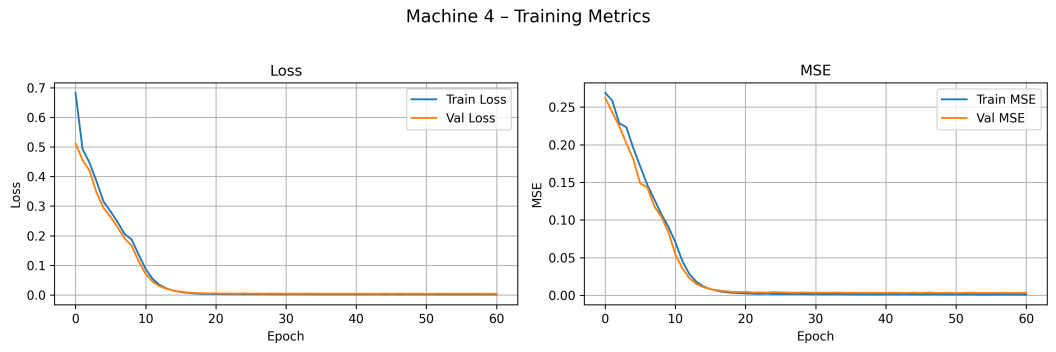


**S1.4.5** – Machine 4 LSTM training history


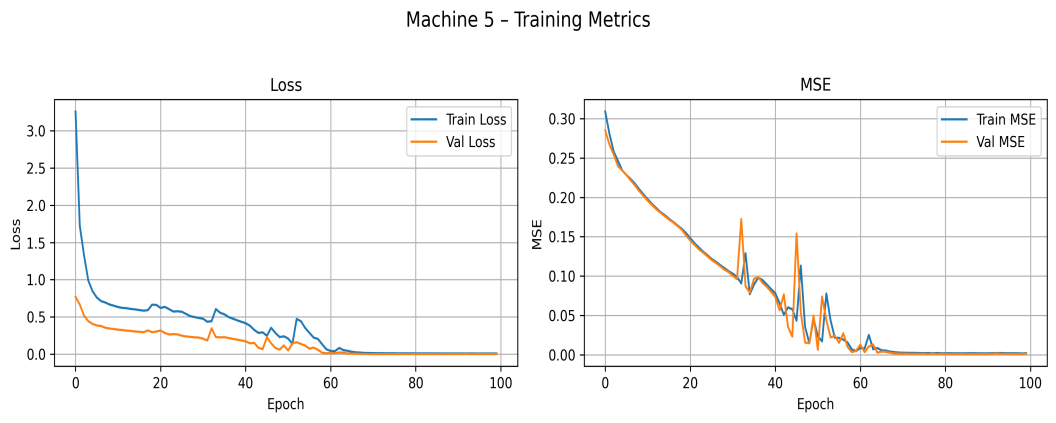


**S1.4.6** – Machine 5 LSTM training history


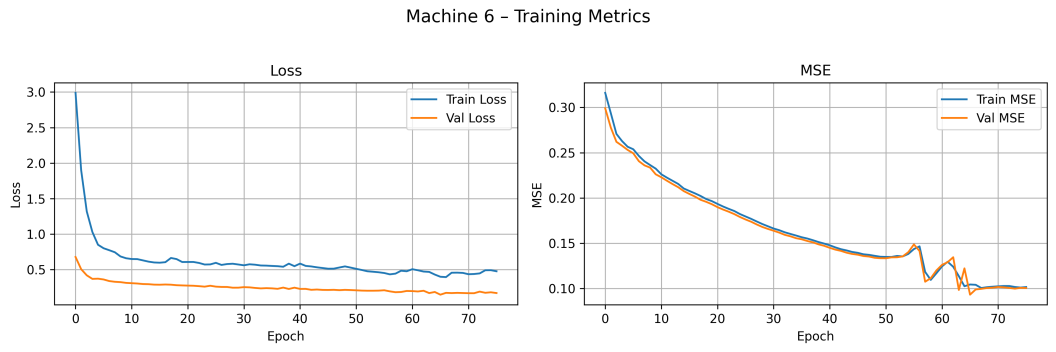


**S1.4.7** – Machine 6 LSTM training history


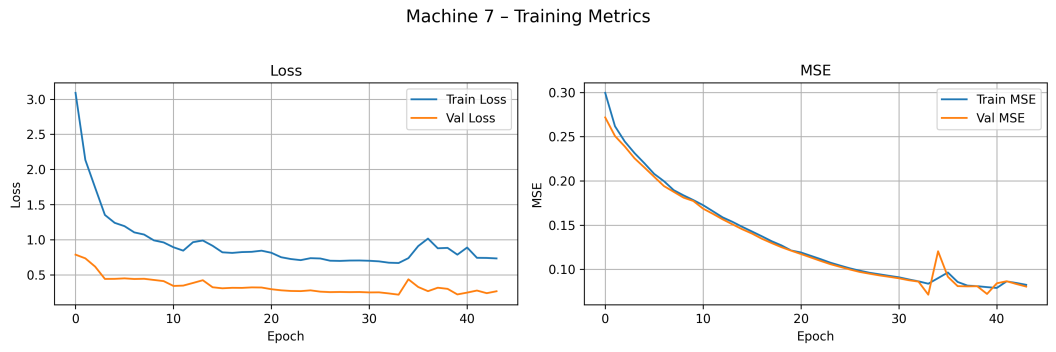


**S1.4.8** – Machine 7 LSTM training history


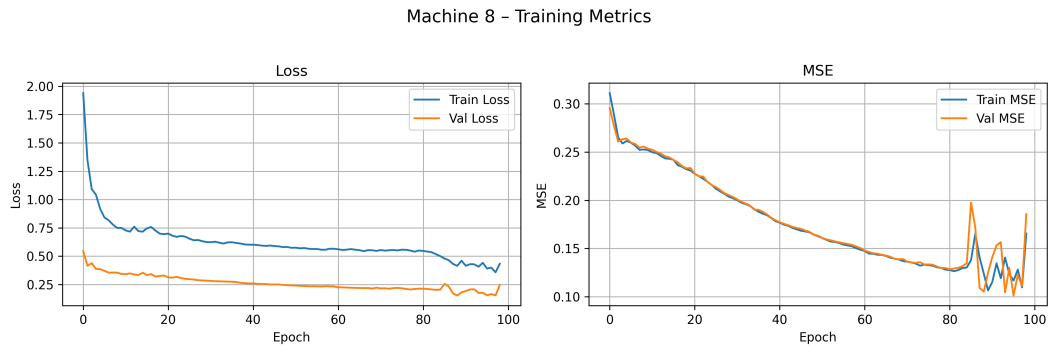


**S1.4.9** – Machine 8 LSTM training history


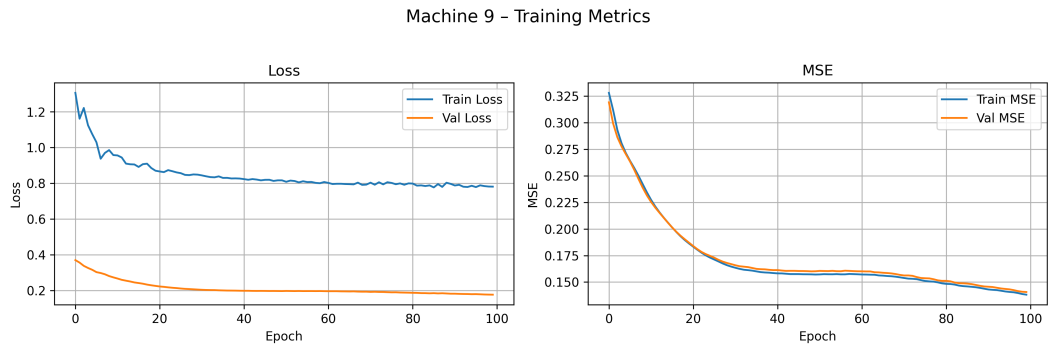


**S1.4.10** – Machine 9 LSTM training history

**S1.4.11** – Machine 10 LSTM training history


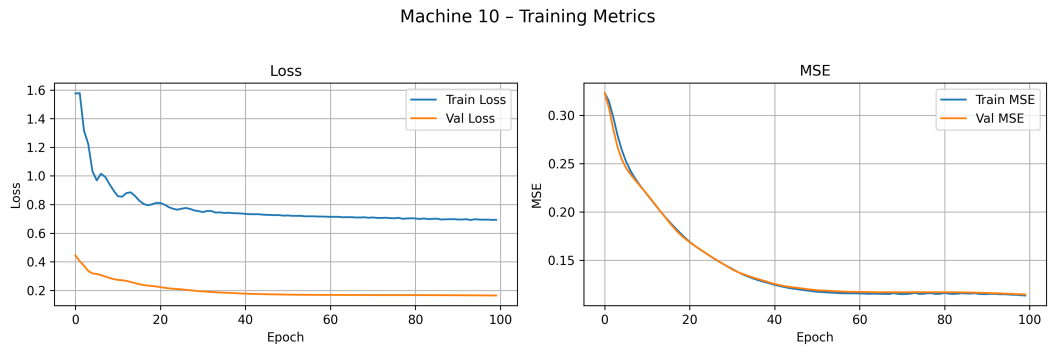


**S1.4.12** – Machine 11 LSTM training history


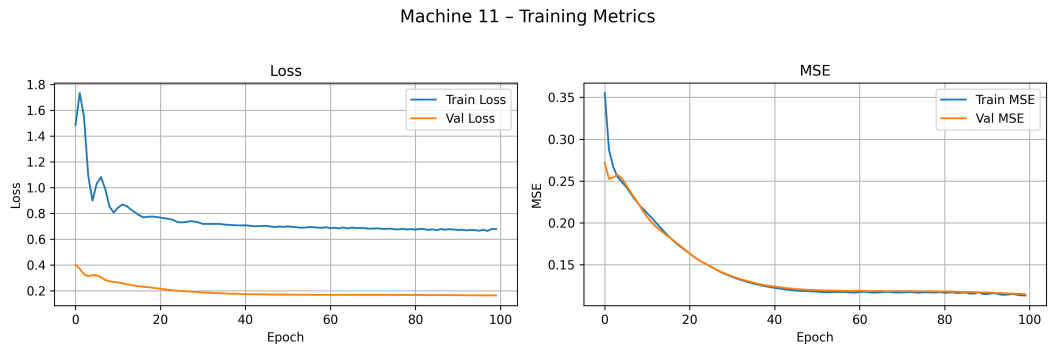


**S1.4.13** – Machine 12 LSTM training history


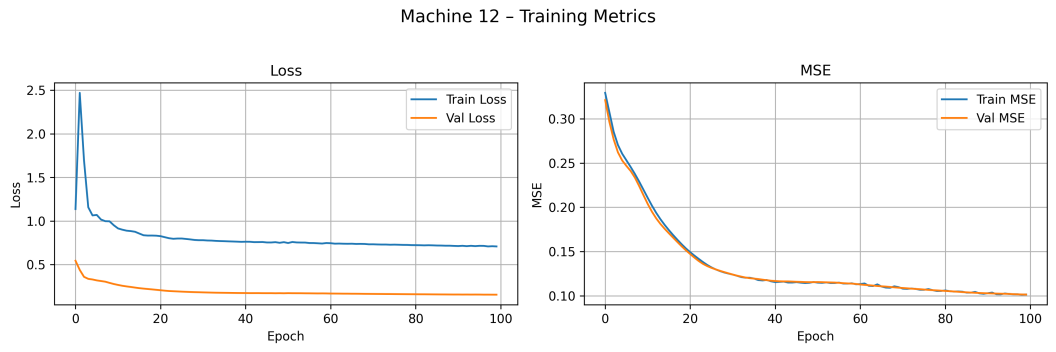


**S1.4.14** – Machine 13 LSTM training history


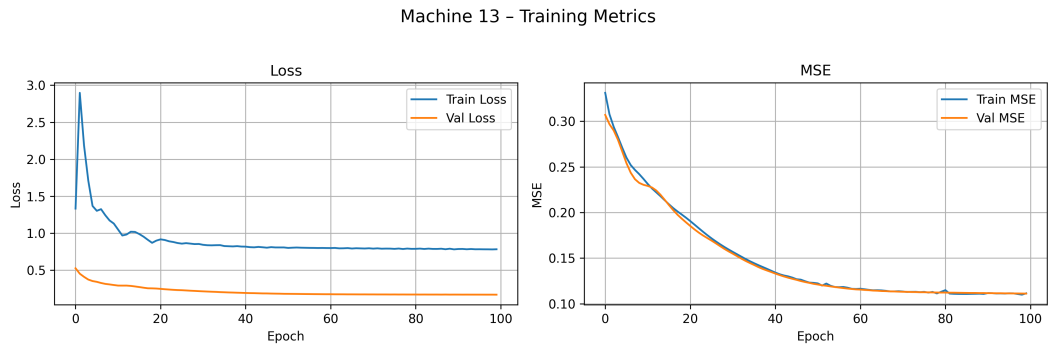


**S1.4.15** – Machine 14 LSTM training history


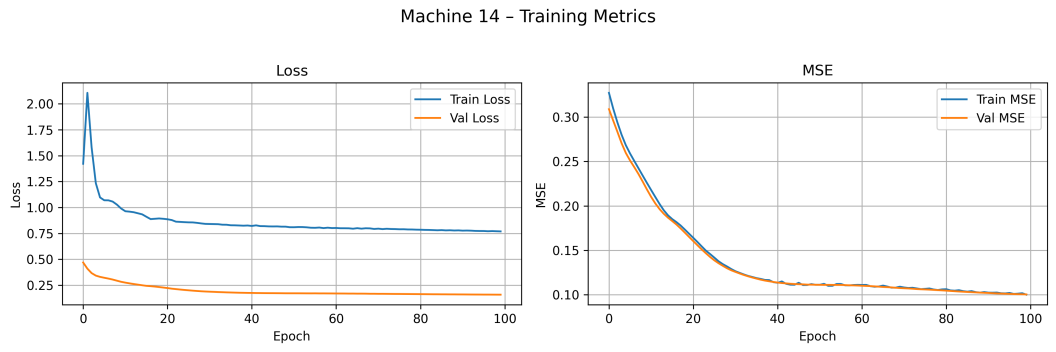


**S1.4.16** – Machine 15 LSTM training history


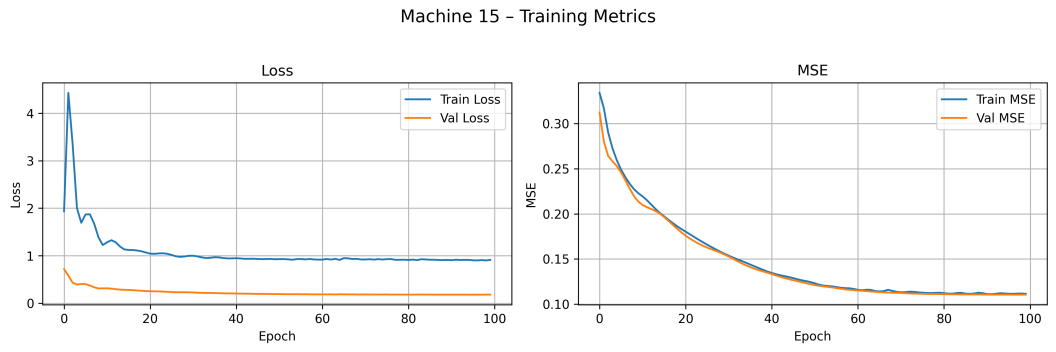


S1.5 List of all tried distributions

| **Distribution** | **Description** |
| --- | --- |
| weibull_min | Classic distribution for modeling time to failure; flexible for  increasing/decreasing hazard. |
| gamma | Flexible skewed distribution; models time to failure or waiting times. |
| lognorm | Models multiplicative effects; right-skewed, positive-only. |
| fatiguelife | Used in mechanical fatigue life modeling; heavy-tailed. |
| invgauss | Inverse Gaussian; models degradation and first-passage time. |
| expon | Memoryless exponential distribution; simplest time-to-event model. |

| gompertz | Common in survival analysis; increasing failure rate over time. |
| --- | --- |

***The loc parameter shifts the distribution to fit negative predicted RUL values, representing cases where the system is predicted to have already failed***

S1.6: Appendices

# Appendix A: WGAN Model Training History

## Model training history for Machine 0: (A) Critic loss,

**(B) Generator loss training epochs.**


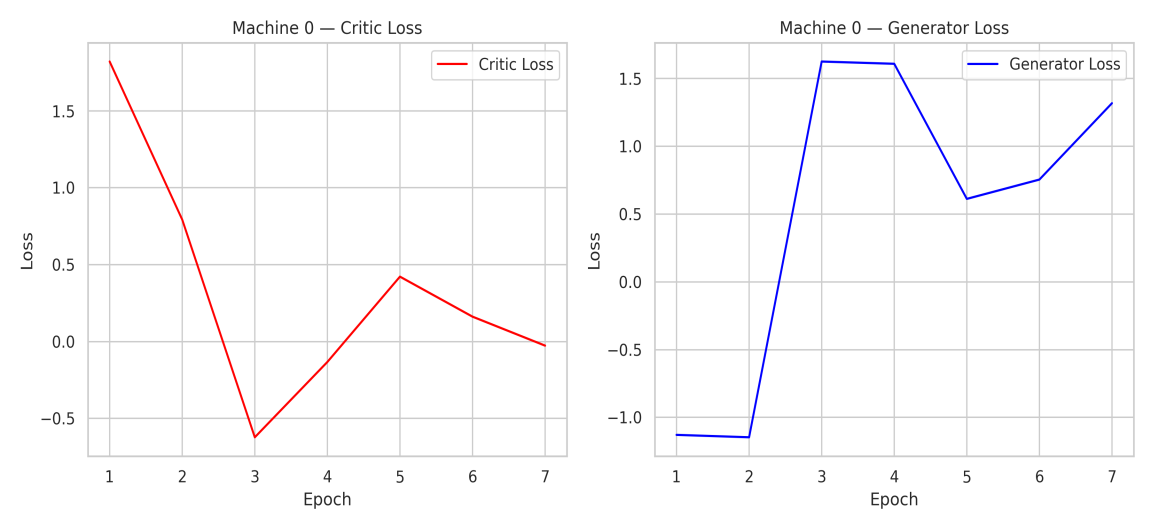


**Fig. A1** Machine 0 training history graphs.

## WGAN Model training history for Machine 1: (A) Critic loss, (B) Generator loss across training epochs.


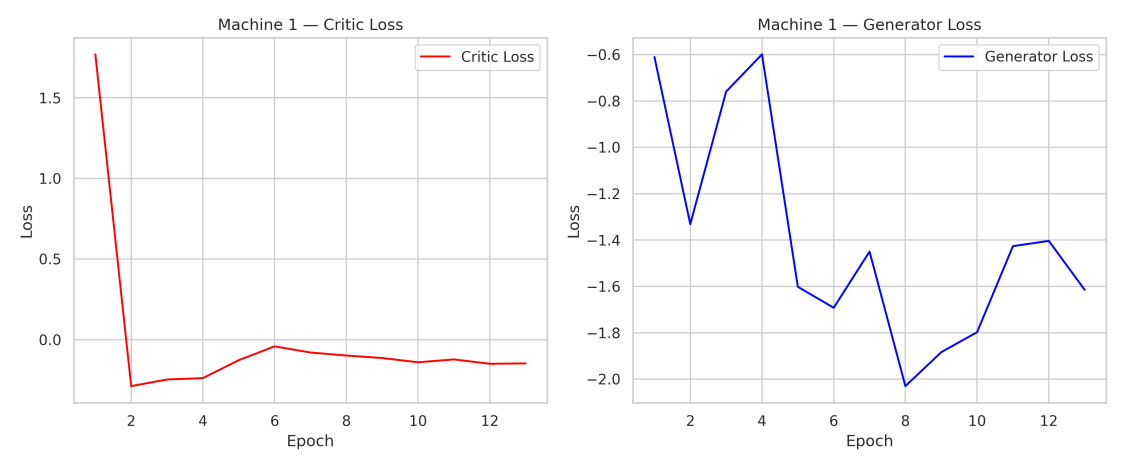


**Fig. A2** WGAN Machine 1 training history graphs.

## WGAN Model training history for Machine 2: (A) Critic loss, (B) Generator loss across training epochs.


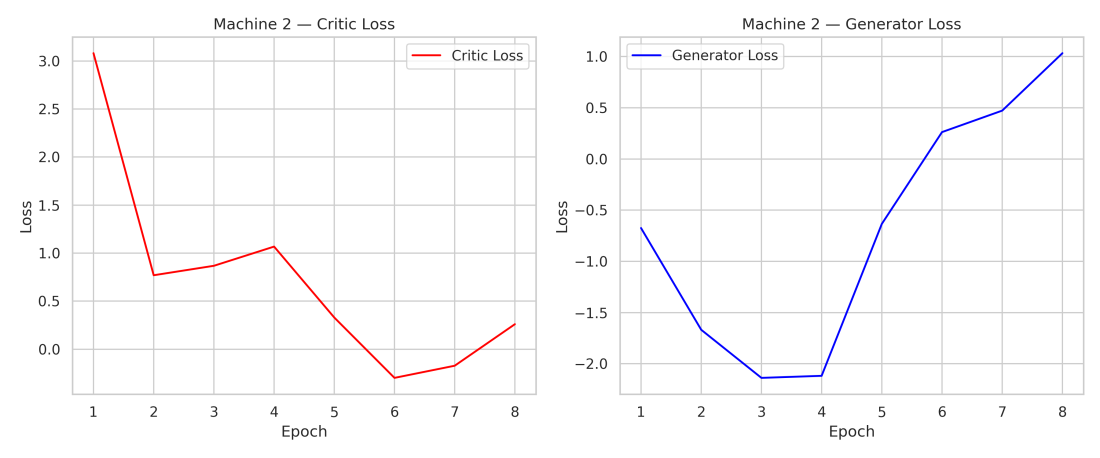


**Fig. A3** Machine 2 training history graphs.

The complete set of corresponding plots for all 16 machines (Machine 0 to Machine 15) is available in Supplementary File S1.

# Appendix B Density-curve comparisons between real and synthetic failure data

## Density-curve comparisons between real and synthetic failure data for Machine 0: (A) Feature 1, (B) Feature 2, and (C) Feature 3


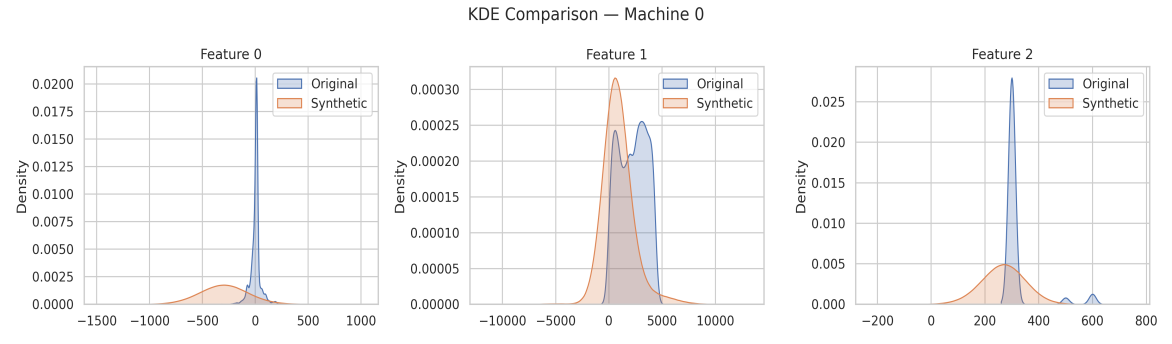


**Fig. B1** Machine 0 density curves.

## Density-curve comparisons between real and synthetic failure data for Machine 1: (A) Feature 1, (B) Feature 2, and (C) Feature 3


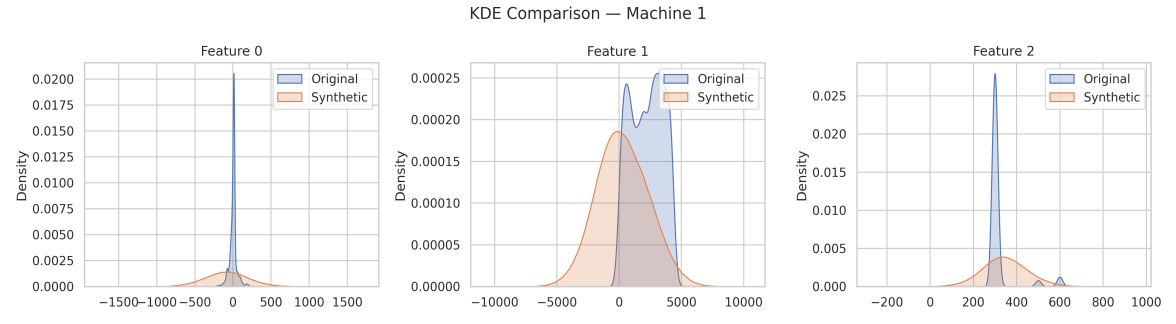


**Fig. B2** Machine 1 density curves.

## Density-curve comparisons between real and synthetic failure data for Machine 2: (A) Feature 1, (B) Feature 2, and (C) Feature 3


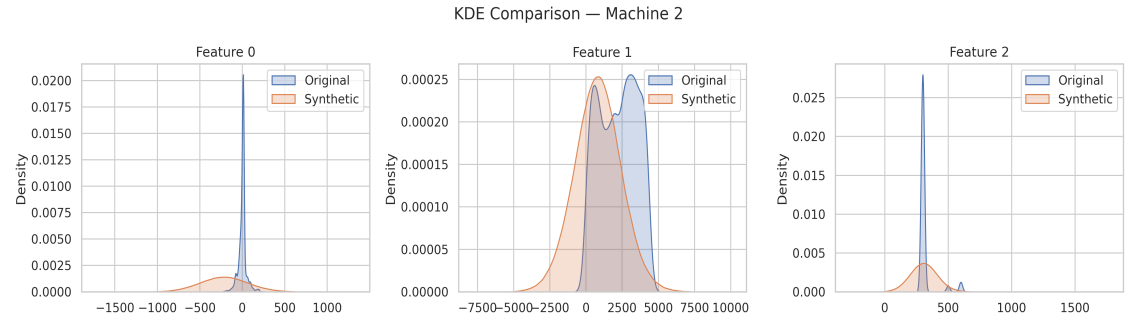


**Fig. B3** Machine 2 density curves.

# Appendix C PCA-Based Comparisons Between Real and Synthetic Data

## PCA comparison between real and synthetic data for Machine 0


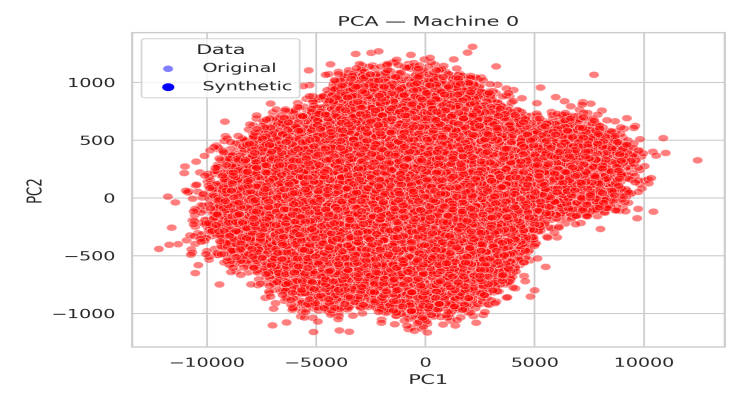


**Fig. C1** PCA plot comparing real and synthetic data for Machine 0. The real and generated sam- ples are projected onto the first two principal components to visualize their distribution in reduced dimensional space. This comparison highlights the structural similarity between the real and syn- thetic datasets.

## PCA comparison between real and synthetic data for Machine 1


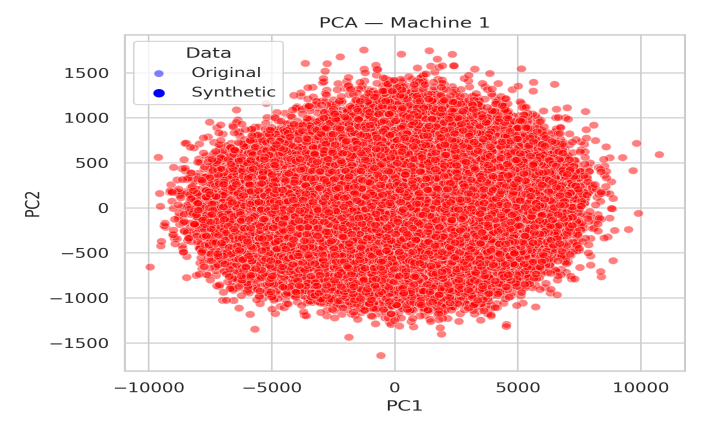


**Fig. C2** PCA plot comparing real and synthetic data for Machine 1. The real and generated samples are projected onto the first two principal components to visualize their distribution in reduced- dimensional space. This comparison highlights the structural similarity between the real and synthetic datasets.

## PCA comparison between real and synthetic data for Machine 2


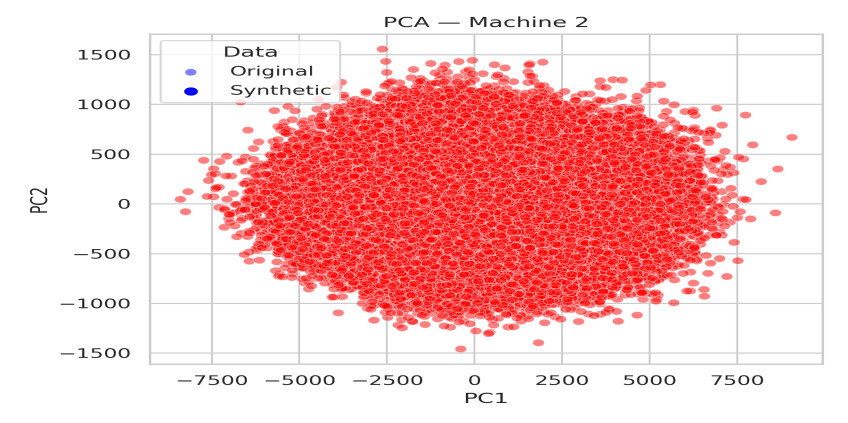


**Fig. C3** PCA plot comparing real and synthetic data for Machine 2. The real and generated samples are projected onto the first two principal components to visualize their distribution in reduced dimensional space. This comparison highlights the structural similarity between the real and syn- thetic datasets.

# Appendix D LSTM model training and validation loss

## LSTM model training and validation loss for Machine


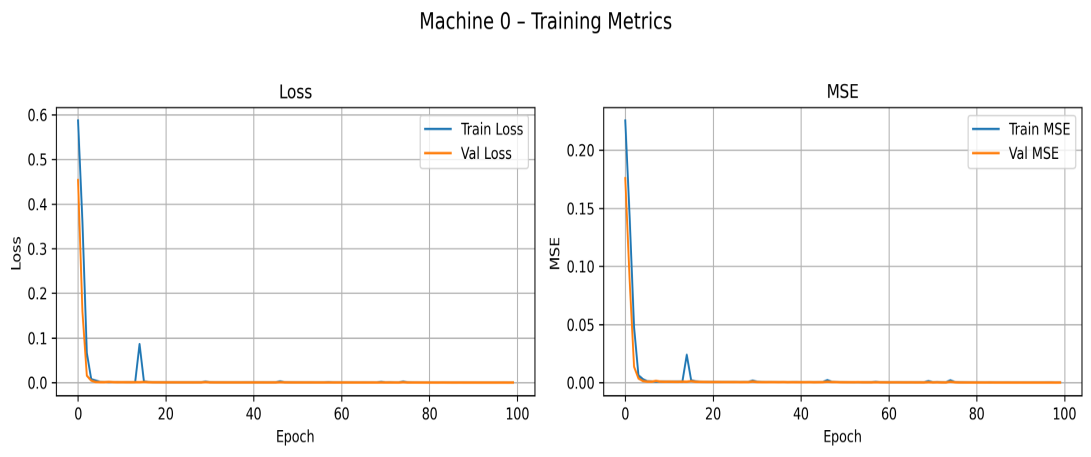


**Fig. D1** LSTM model training and validation loss for Machine 0

## LSTM model training and validation loss for Machine 1


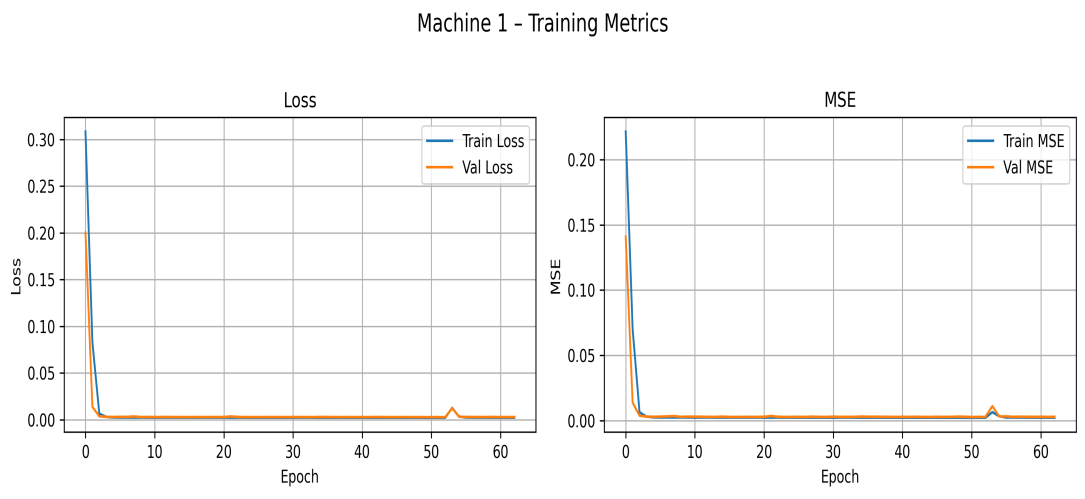


**Fig. D2** LSTM model training and validation loss for Machine 1.

## LSTM model training and validation loss for Machine 2


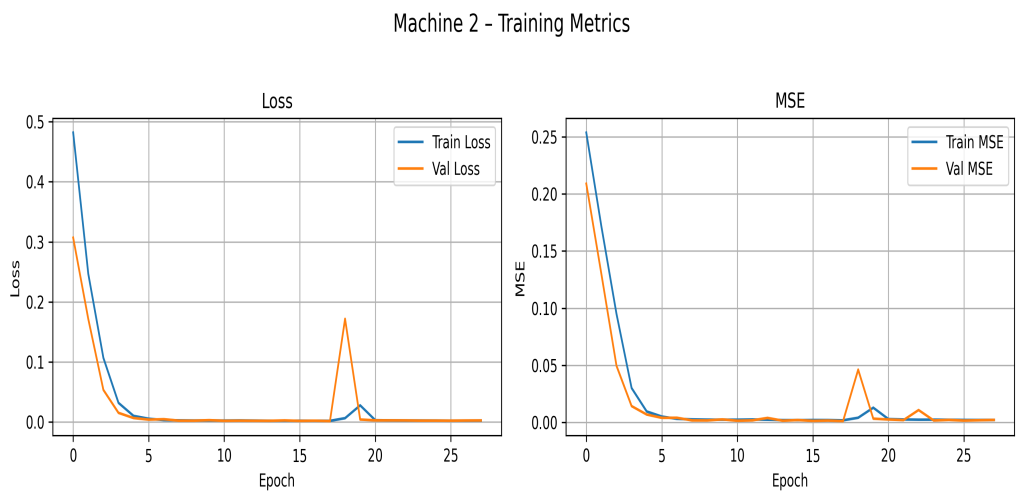


**Fig. D3** LSTM model training and validation loss for Machine 2.

# Appendix E Experimenting different strategies with validation data

## E.1 : Trend in total costs across survival thresholds


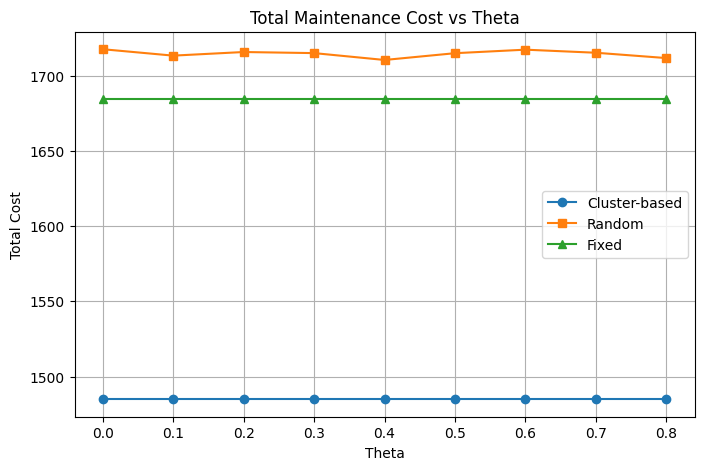


## E.2 : Average costs across different strategies


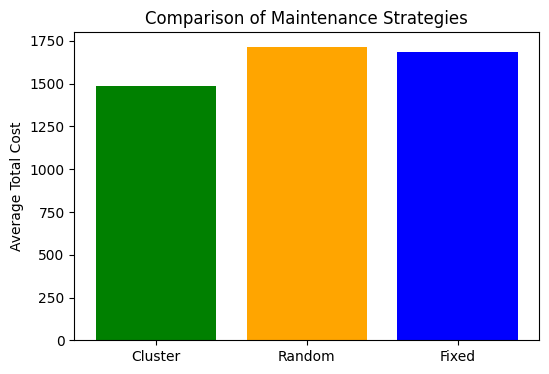


## E.3 : Trend in number of maintenance practices across survival thresholds


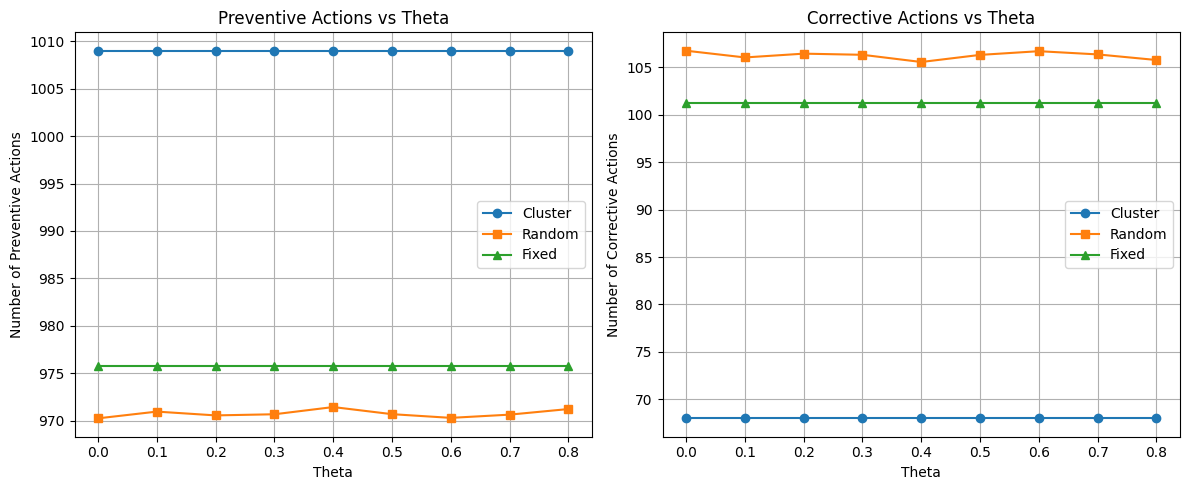


#### References
